# Formation of Carbon-Induced Nitrogen-Centered Radicals on Titanium Dioxide under Illumination

**DOI:** 10.1021/jacsau.3c00556

**Published:** 2023-11-27

**Authors:** Po-Wei Huang, Nianhan Tian, Tijana Rajh, Yu-Hsuan Liu, Giada Innocenti, Carsten Sievers, Andrew J. Medford, Marta C. Hatzell

**Affiliations:** † School of Chemical & Biomolecular Engineering, Georgia Institute of Technology, Atlanta, Georgia 30332, United States; ‡ School of Molecular Science, Arizona State University, Tempe, Arizona 85281, United States; § Center of Nanoscale Materials, Argonne National Laboratory, Woodridge, Illinois 60517, United States; ∥ School of Civil and Environmental Engineering, Georgia Institute of Technology, Atlanta, Georgia 30332, United States; ⊥ George W .Woodruff School of Mechanical Engineering, Georgia Institute of Technology, Atlanta, Georgia 30332, United States

**Keywords:** Photocatalysis, Carbon−Nitrogen Bond, Ammonia, Nitrogen Fixation, Titania

## Abstract

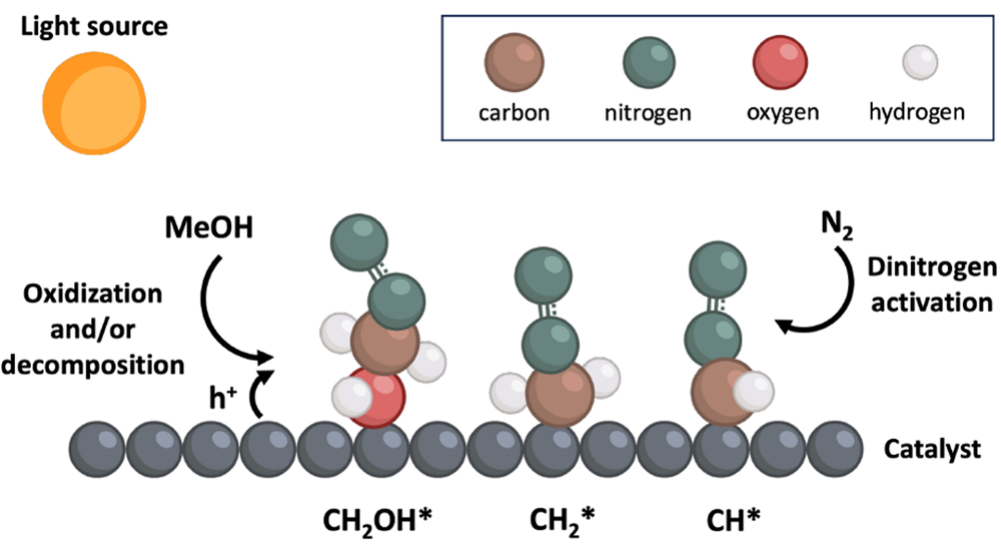

Titanium dioxide is the most studied photocatalytic material and has been reported to be active for a wide range of reactions, including the oxidation of hydrocarbons and the reduction of nitrogen. However, the molecular-scale interactions between the titania photocatalyst and dinitrogen are still debated, particularly in the presence of hydrocarbons. Here, we used several spectroscopic and computational techniques to identify interactions among nitrogen, methanol, and titania under illumination. Electron paramagnetic resonance spectroscopy (EPR) allowed us to observe the formation of carbon radicals upon exposure to ultraviolet radiation. These carbon radicals are observed to transform into diazo- and nitrogen-centered radicals (e.g., CH_*x*_N_2_^•^ and CH_*x*_NH_*y*_^•^) during photoreaction in nitrogen environment. In situ infrared (IR) spectroscopy under the same conditions revealed C–N stretching on titania. Furthermore, density functional theory (DFT) calculations revealed that nitrogen adsorption and the thermodynamic barrier to photocatalytic nitrogen fixation are significantly more favorable in the presence of hydroxymethyl or surface carbon. These results provide compelling evidence that carbon radicals formed from the photooxidation of hydrocarbons interact with dinitrogen and suggest that the role of carbon-based “hole scavengers” and the inertness of nitrogen atmospheres should be reevaluated in the field of photocatalysis.

Photochemical nitrogen transformations were initially proposed by N. R. Dhar, a prominent Indian soil scientist in the 1920–1930s.^1^ These chemical transformations were investigated within a broader discussion on the role of radiation theory in chemical reactions.^2,3^ Although radiation theory was largely disproven, the experimental observations during this period provided a pathway toward the modern understanding of photocatalysis. Specifically, Dhar’s early work focused on illuminating soils that contained minerals and differing concentrations of hydrocarbons. In these early works, the level of abiotic ammonia synthesis was reported to increase in the presence of sunlight and hydrocarbons. Others continued to evaluate this process;^4^ however, these results remained unverified in a controlled setting until 1977, when Schrauzer and Guth independently reported photocatalytic nitrogen fixation with titania in a modern photochemical setup.^5,6^ Despite the long history of this reaction, the reproducibility of results is still not well-established,^7−10^ and molecular-scale mechanistic insight is still limited.

Since these early investigations, there has been growing interest in understanding photocatalytic nitrogen fixation with a range of photocatalysts.^10−15^ There are three dominant hypotheses regarding the way nitrogen is converted to ammonia. The first suggests that nitrogen fixation occurs through a direct reduction process (electron-driven) at or near an oxygen vacancy site on titania;^10,12−15^ this is the hypothesis assumed in the vast majority of studies, although the position of the conduction band edge of titania yields a low overpotential (∼0.1 V) that is unlikely to be sufficient to overcome kinetic barriers. The second hypothesis suggests that oxidative processes (driven by holes) play a role through photolysis^16^ or nitrogen oxidation.^17^ Although this hypothesis would result in a much higher driving force from the strong oxidative potential of photogenerated holes, there is still the issue of generally weak adsorption of N_2_ to oxygen vacancies (*E*_ads_ ≈ 0.2 eV)^18^ and a lack of direct experimental evidence supporting the role of hydroxyl or nitrogen oxide intermediates. The third hypothesis, recently proposed by some authors, suggests that photogenerated carbon radical species mediate the reaction.^18,19^ Theoretical calculations revealed that carbon radicals can interact strongly with nitrogen (*E*_ads_ ≈ −1.89 eV) and relatively selectively, and ambient pressure X-ray photoelectron spectroscopy revealed the presence of surface nitrogen only when surface carbon was present.^18^

The hypothesis that carbon species can react with dinitrogen is broadly consistent with findings from combustion chemistry,^20^ homogeneous catalysis,^21^ and some recent results in electrocatalysis.^22,23^ In combustion chemistry, carbon radicals, such as CH^•^, are known to activate nitrogen through the formation of diazo species.^20^ The formation of C–N bonds has also been shown to be a viable route to activating dinitrogen in the coordination chemistry of homogeneous catalysts,^21^ and recent work on direct electrocatalytic urea synthesis has indicated that carbon species, such as CO, can promote the electrocatalytic dissociation of the nitrogen bond.^22,23^ However, the interactions between carbon radicals and nitrogen in photocatalysis have not been widely examined. Besides influencing photocatalytic ammonia synthesis, these C–N interactions could impact the interpretation of many other photocatalytic experiments because they suggest that carbonaceous hole scavengers may serve as cocatalysts or reactants and indicate that the assumption of nitrogen environments being chemically inert should be re-examined in photocatalytic experiments involving carbon.

Here, we used various spectroscopic techniques to investigate the interaction between carbon radicals and dinitrogen on illuminated titania. We conducted electron paramagnetic resonance (EPR) measurements to observe the formation of radicals (e.g., diazo- and nitrogen-centered radicals) during photocatalytic processes and to identify intermediates during the interaction of radicals with nitrogen. We then used in situ infrared (IR) spectroscopy to identify functional groups on the catalyst surface during the photocatalytic reaction. Finally, DFT simulations were employed to evaluate the ground-state free energies of intermediate states to identify thermodynamic barriers and the most stable molecular structures with and without carbon. The results reveal consistent evidence that carbon radicals interact with dinitrogen on the illuminated titania surfaces.

## Results and Discussion

**Figure 1:**
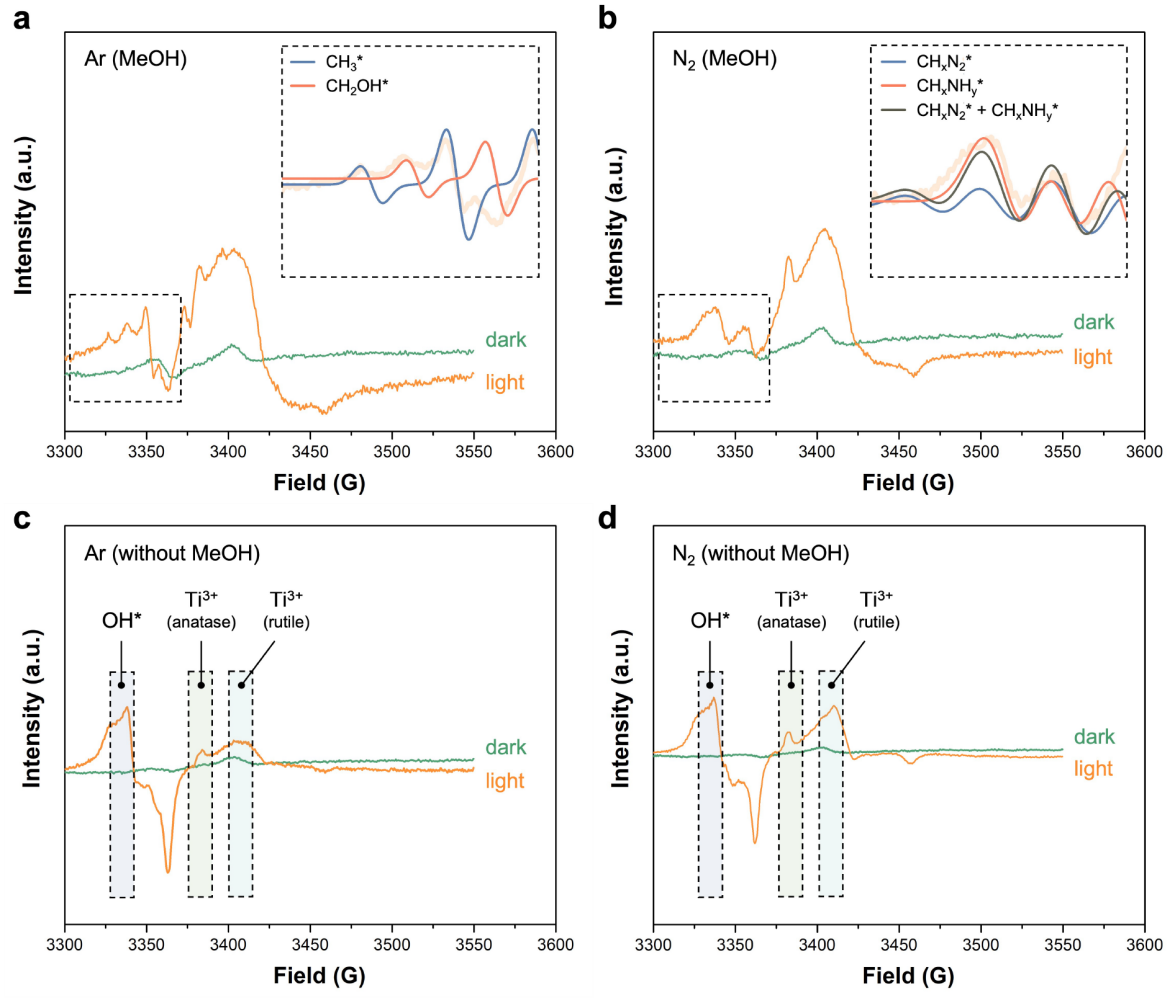
(a) EPR spectra of titania with 5 vol % methanol solution at 4.2 K under argon environment with and without illumination. (b) EPR spectra of titania with 5 vol % methanol solution at 4.2 K under nitrogen environment with and without illumination. (c) EPR spectra of titania without methanol at 4.2 K under argon environment with and without illumination. (d) EPR spectra of titania without methanol at 4.2 K under nitrogen environment with and without illumination.

Electron paramagnetic resonance (EPR) and infrared (IR) spectroscopy are two approaches that can probe reaction intermediates in the initial stages of charge separation. EPR was specifically used to track organic radicals that may form under illumination. The EPR experiments were first conducted using titania as the catalyst in the presence of methanol (a commonly used carbonaceous hole scavenger in photocatalysis) in an argon environment (Figure 1a). In the absence of light, no signals were observed. Conversely, under illumination, the EPR spectra showed the emergence of a methyl radical (CH_3_^•^) and a hydroxymethyl radical (CH_2_OH^•^). The EPR spectrum in the region of *g* = 2.004 that corresponds to organic radicals is composed of a quartet spectrum characteristic of methyl radicals with intensity ratios of 1:3:3:1 and hyperfine splitting constant of *A*_H_ = 23*G*^24^ and a triplet radical characteristic of hydroxymethyl radical with intensities 1:2:1 and *A*_H_ = 20*G* hyperfine coupling.^25^ Both radicals are well-documented products of the methanol oxidation reaction that occurs with the aid of the photogenerated holes.^26^ When the argon environment is replaced with nitrogen gas, the methyl radicals transform into diazo radicals (CH_*x*_N_2_^•^) with the quintet EPR spectrum with line intensities 1:3:5:3:1 and *A*_N_ = 19*G* hyperfine coupling^27,28^ and single nitrogen-centered radicals (CH_*x*_NH_*y*_^•^) exhibiting a characteristic triplet spectrum of an I=1 atom with *A*_N_ = 15*G* hyperfine coupling (Figure 1b).^29−31^ Single nitrogen-centered radicals (CH_*x*_NH_*y*_^•^) could potentially be methylamine radicals (CH_3_NH^•^); however, because of the line broadening at the surface of titania, we were unable to observe additional secondary hyperfine splitting originating from the added hydrogen atom. Similar radical formation is also observed when the nitrogen gas (^14^N_2_) is replaced with isotope-labeling ^15^N_2_ gas (Figure S3).

**Figure 2:**
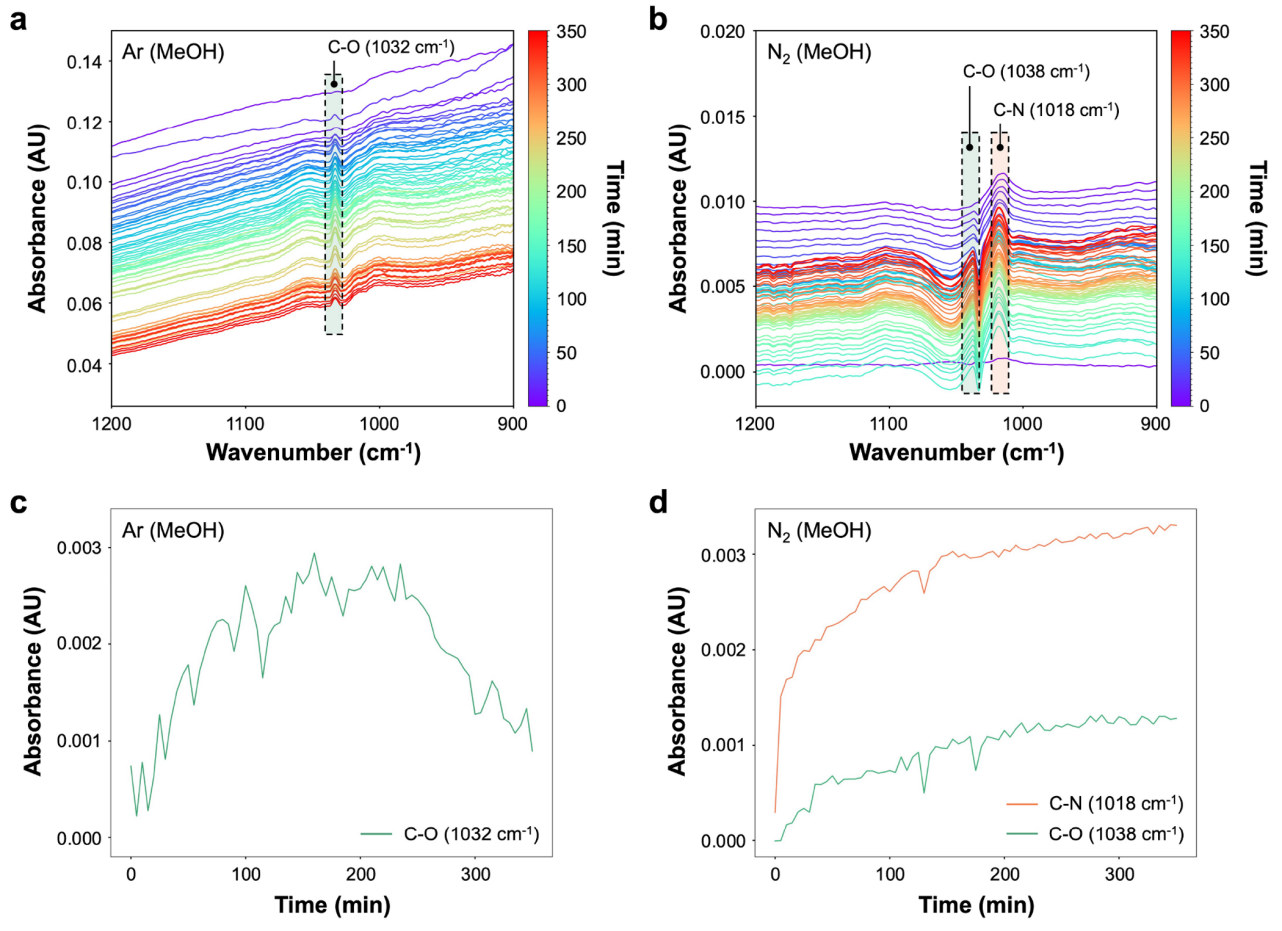
IR spectra of titania with 5 vol % methanol solution (a) under argon environment and (b) nitrogen environment during illumination. Trends of intensities with time of targeted wavenumbers under (c) argon environment and (d) nitrogen environment during illumination.

To further confirm the interaction of carbon with nitrogen, we probed surface species with in situ infrared (IR) spectroscopy using a customized ATR flow cell (Figure S1). In the presence of methanol, the IR spectra showed an obvious C–N stretching band (1018 cm^–1^) on the titania surface under the nitrogen environment during illumination, while no C–N stretching band appeared under the argon environment (Figure 2a,b). Both spectra contained a C–O stretching band (1032 and 1038 cm^–1^) that is attributed to methanol. The evolution of the IR spectra demonstrated an increase in the intensity of the C–N stretching band with increasing illumination time (Figure 2d). Control IR experiments were conducted in the absence of methanol; no C–N stretching or C–O stretching bands were observed in either argon or nitrogen environments under illumination (Figure S4). Additionally, the formation of the C–N stretching band is not attributed to alternative reactions, such as the condensation of NH_3_ (a photoreduction product of nitrogen) and HCHO (a photooxidation product of methanol). NH_3_ is expected to bind strongly to the surface, and HCHO, a well-known hole scavenger, if formed during the reaction, efficiently reacts with photogenerated holes, which makes the condensation of HCHO and NH_3_ unlikely under photocatalytic conditions.^32^ That is, the observed formation of the C–N stretching band supports the results obtained from EPR, thereby indicating the formation of diazo species (CH_*x*_N_2_^•^) and/or single nitrogen-centered radicals (CH_*x*_NH_*y*_^•^) and further corroborates the interaction of carbon with nitrogen on titania under illumination.

**Figure 3:**
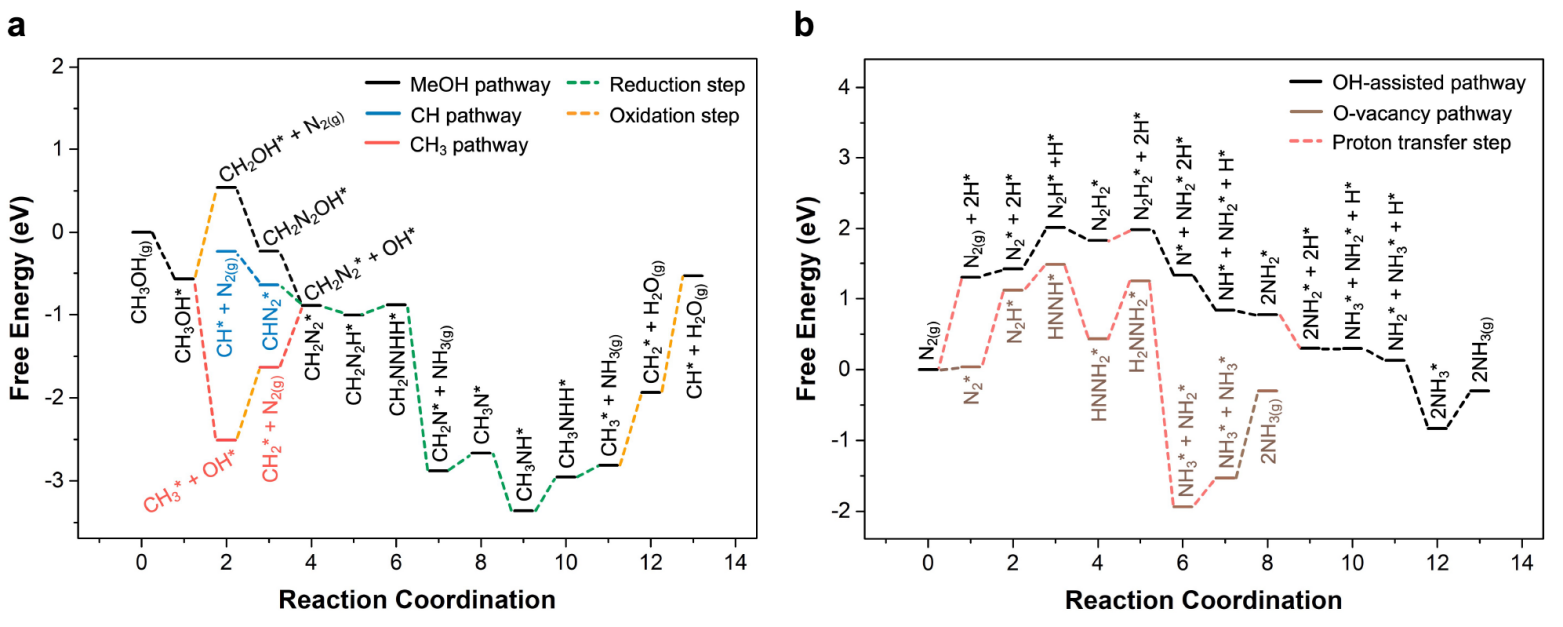
(a) Free energy diagram of photocatalytic nitrogen fixation on titania with the existence of methanol. (b) Free energy diagram of photocatalytic nitrogen fixation on titania without methanol. The thermodynamic barrier is calculated as the largest energy barrier along the reduction pathway. Thermodynamic barriers for different mechanisms: 0.55 eV (from step 9 to step 11 for the carbon-assisted mechanism); 1.48 eV (from step 0 to step 3 for the oxygen vacancy mechanism); 2.01 eV (from step 0 to step 3 for the OH-assisted mechanism). Note that oxidative steps are not included in this calculation, as they have a large driving force due to the strong oxidizing potential of photogenerated holes on titania, and the desorption of NH_3_ is also excluded, assuming it is in equilibrium with desorbed NH_3_.

Density functional theory (DFT) simulations are employed to evaluate the energetics of carbon–dinitrogen interactions and various mechanisms for carbon-assisted nitrogen fixation on titania. The P25 titania catalyst used in the experimental sections is polycrystalline with both anatase and rutile phases and multiple exposed facets. Prior single-crystal surface science experiments and calculations revealed carbon–nitrogen coupling on the rutile (110) facet, so we select it as a model surface.^17^ Calculations are done with a combination of BEEF-vdW^33^ and HSE06^34^ functionals, as detailed in the Supporting Information. The free energy diagram for methanol decomposition and the interaction of CH^*^ and CH_3_^*^ intermediates with nitrogen is shown in Figure 3a at 0 V vs RHE, where the computational hydrogen electrode is used for all proton/electron transfer steps.^35^ The formation of the CH_2_OH^*^ intermediate is thermodynamically uphill under neutral conditions, but becomes favorable under oxidizing conditions and is known to occur because of the observation of CH_2_OH^•^ radicals in EPR (Figure 1a). The interaction of these hydroxymethyl species with N_2_ results in a strongly exothermic reaction to form CH_2_N_2_^*^, which suggests that this is the primary interaction between carbon radicals and N_2_. Once the CH_2_N_2_^*^ intermediate is formed, subsequent hydrogenations are primarily downhill or have surmountable thermodynamic barriers of ≤0.55 eV.^36^ This reaction mechanism reveals a thermodynamically feasible route for the reaction of methanol to form diazo radicals (CH_*x*_N_2_^•^), as well as the formation of single nitrogen-centered radicals (CH_x_NH_y_^•^) and other compounds with C–N bonds.

The interaction between methanol and N_2_ is not catalytic as there is no route to regenerate the methanol precursor. However, the free energy diagram shows that the reaction between adsorbed CH^*^ and N_2_ is also exergonic, similar to the mechanism where CH^•^ radicals activate N_2_ gas in combustion chemistry.^20^ The CH^*^ mechanism details with the CH_2_N_2_^*^ mechanism through exergonic steps, and CH^*^ can, in principle, be regenerated by photooxidation of CH_3_^*^ to CH^*^, thereby closing the catalytic cycle. This mechanism is consistent with the hypothesis that hydrocarbon contamination can facilitate photocatalytic ammonia synthesis^18^ and indicates that methanol may not be required for a carbon-assisted photocatalyic ammonia cycle. Rather, methanol may increase the rate directly by acting as a reactant or indirectly by increasing the concentration of the surface hydrocarbons. However, these results present only a thermodynamically plausible pathway, and the more detailed models required to assess the kinetics, excited states, and competing reactions are beyond the scope of this work.

In contrast, in the system without the presence of methanol, the EPR spectra of titania in both the argon and nitrogen environments under illumination showed only surface OH^•^ radicals and the Ti^3+^ signal, while no significant signals appeared in the dark spectra in either case (Figure 1c,d). The free energy diagram of the photocatalytic fixation of nitrogen on the oxygen vacancy of titania demonstrated a large thermodynamic barrier of 1.48 eV during the reaction, and the hydroxyl-assisted pathway reveals an even larger thermodynamic barrier of 2.01 eV (Figure 3b).^16^ Both of these values are higher than the 0.55 eV barrier in the carbon-assisted pathways (Figure 3a), which demonstrates that the presence of carbon sources offers a more thermodynamically feasible route for the photocatalytic nitrogen fixation reaction through reactions with carbon intermediates.

On the basis of evidence from spectroscopic experiments and DFT, we hypothesize specific reaction mechanisms by which carbon radicals react with dinitrogen on titania. Specifically, CH_2_OH^•^ radicals are generated from the decomposition of methanol, or CH_3_^•^ radicals are oxidized into adsorbed CH^*^ by the photogenerated holes from the titania (Figure 3a). Then, CH_2_OH^•^ radicals or adsorbed CH^*^ combine with nitrogen to form CH_2_N_2_^*^, which is hydrogenated to the CH_2_N_2_H_2_ species, and ammonia can be formed by further hydrogenation. Subsequently, CH_2_N^*^ is protonated to form CH_3_NH_2_^*^, which is hydrogenated and dissociated to form another ammonia molecule. This mechanism relies on eight or more electron transfers and is, thus, not expected to occur at a high efficiency. Moreover, the strong adsorption of NH_3_ indicates that concentrations in the bulk solution will be very low unless washing procedures are applied. However, direct observation of carbon–nitrogen species, along with the theoretical free energy pathway, makes this the most plausible molecular-scale mechanism for the photocatalytic ammonia synthesis on titania. Future work will focus on carefully quantifying the rates and efficiencies of photocatalytic ammonia synthesis using isotopic labeling and additional spectroscopic techniques.

In conclusion, strong evidence obtained through spectroscopic experiments and DFT calculations provides experimental and theoretical support for reactions between photogenerated carbon radicals and molecular nitrogen. EPR spectra revealed a transformation of the methyl radical (CH_3_^•^) and hydroxymethyl radical (CH_2_OH^•^) into diazo radicals (CH_*x*_N_2_^•^) and single-nitrogen-center radicals (CH_*x*_NH_*y*_^•^) during the photocatalytic reaction, and an obvious C–N stretching peak in the IR spectra could only be observed in the presence of methanol and nitrogen under illumination. DFT results reveal that carbon-assisted pathways have thermodynamic barriers of 0.55 eV and suggest a specific molecular-scale mechanism for how methanol and its derivatives react with nitrogen to form ammonia. While the results here focus on titania, we hypothesize that many other semiconductors that have redox properties to oxidize organics also form similar carbon radical species that react with dinitrogen, which provides a new perspective on existing photocatalytic results and suggests additional strategies for photocatalytic dinitrogen activation. These findings also suggest that the inertness of N_2_ and the role of carbonaceous “hole scavengers” should be broadly re-examined in the field of photocatalysis, particularly in the context of nitrogen chemistry.

## Methods

### Electron Paramagnetic Resonance

Continuous wave electron paramagnetic resonance (CW-EPR) spectra were acquired using a Bruker ELEXYS E500 spectrometer operating at X-band (9.4 GHz) frequencies with an Oxford ESR900 He flow cryostat with an ITC-5025 temperature controller and a Bruker High QE (HQE) cavity resonator (ER 4122SHQE). g tensors were calibrated for accuracy using known BDPA (α,γ-bisdiphenylene-β-phenylallyl) and Mn^2+^ in SrO standards. The acquisition parameters, such as the receiver gain, modulation amplitude, and microwave power, were all optimized and then kept constant to enable the comparison of multiple samples under the same conditions. The modulation amplitude was set to 5*G*, and the microwave power was 0.5 mW unless otherwise stated. Ten mg of P25 titania was added into 0.1 mL of DI water. Five vol % of methanol was added as a presence of carbon source. Argon or nitrogen were saturated into the tube for 40 min, and a 300 W xenon lamp was used as the illumination source for the light samples. The samples were sealed under He, and the EPR spectra were recorded at 4.2 K. All spectra were simulated using EasySpin software package Release 6.0.0-dev.26 (2020-10-12) (Stefan Stoll, Arthur Schweiger, EasySpin, a comprehensive software package for spectral simulation and analysis in EPR).^37^

### In Situ Infared Spectrascopy

In situ ATR measurements were performed using a Thermo Fisher iS20 instrument equipped with an MCT/A detector. The Pike Technologies HATR accessory was used to investigate the reaction on the catalyst surface. The top of the HATR cell was modified in house with the addition of a quartz window to perform photocatalytic experiments. In a typical experiment, a slurry was prepared by adding 2 mL of water to 15 mg of P25 and vigorously mixed until well dispersed. The slurry was then deposited dropwise on a Ge refractive element (IRE), and the water was evaporated with a heat gun. The coated IRE was inserted into the modified HATR module. A second IR spectrometer (Thermo Fisher iS5) equipped with a HATR cell using a ZnSe crystal was used to analyze the effluent of iS20 to account for the contribution of the liquid phase. The solution of 5 vol % of MeOH was saturated with the gas of interest (Ar or N_2_) for 40 min before being fed into the system. The solution was continuously purged with the gas for the entire length of the experiment to maintain saturation. The methanol solution was fed into the system at 0.5 mL min^–1^ for 30 min to stabilize the flow. Afterward, the xenon lamp was turned on at 300 W. The acquisition of IR spectra was started on both the iS20 and the iS5, and the evolution of the spectra was followed for 350 min with collection of a spectrum every 5 min by using a resolution of 4 cm^–1^ and 64 scans. The last spectrum collected in the absence of irradiation was used as background and subtracted from the spectra obtained in the presence of irradiation to highlight the vibrations of the molecules bound to the catalyst surface.

## Supporting Information Available

The material is available and free of charge via the Internet at https://pubs.acs.org/ The Supporting Information is available free of charge at https://pubs.acs.org/doi/10.1021/jacsau.3c00556.

Detailed experiments, including EPR, in situ IR, and density functional theory calculations (PDF)
