# Supplementary material for: Formation of Carbon-Induced Nitrogen-Centered Radicals on Titanium Dioxide under Illumination
Source: JACS Au. 2023 Nov 27;3(12):3283–9. doi: 10.1021/jacsau.3c00556 (PMC10751760; doi:10.1021/jacsau.3c00556)
Supplement: Supplementary file 1 — au3c00556_si_001.pdf [file au3c00556_si_001.pdf]

Supporting information for: Formation of carbon-induced nitrogen-centered radicals on titanium dioxide under illumination

Po-Wei Huang,† Nianhan Tian,† Tijana Rajh,‡ Yu-Hsuan Liu,§ Giada Innocenti,† Carsten Sievers,† Andrew J. Medford,*,† and Marta C. Hatzell*,‖

†*School of Chemical and Biomolecular Engineering, Georgia Institute of Technology, Atlanta, GA, 30332 USA*

‡*School of Molecular Science, Arizona State University, Tempe, AZ, 85281 USA*

¶*Center of Nanoscale Materials, Argonne National Laboratory, Woodridge, IL, 60517 USA*

§*School of Civil and Environmental Engineering, Georgia Institute of Technology, Atlanta, GA, 30332 USA*

‖*Woodruff School of Mechanical Engineering, Georgia Institute of Technology, Atlanta, GA, 30332 USA*

E-mail: ajm@gatech.edu; marta.hatzell@me.gatech.edu

Experimental Methods

Electron Paramagnetic Resonance

Continuous wave electron paramagnetic resonance (CW-EPR) spectra were acquired using a Bruker ELEXYS E500 spectrometer operating at X-band (9.4 GHz) frequencies with an Oxford ESR900 He flow cryostat with an ITC-5025 temperature controller and a Bruker

High QE (HQE) cavity resonator (ER 4122SHQE). g tensors were calibrated for accuracy using known BDPA and Mn^{2+} in SrO standards. The acquisition parameters such as the receiver gain, modulation amplitude, and microwave power were all optimized and then kept constant to enable comparison of multiple samples under the same conditions. The modulation amplitude was set to 5G and the microwave power was 0.5 mW unless otherwise stated. 10 mg of P25 titania were added into 0.1 mL of DI water. 5 vol% of methanol were added as a presence of carbon source. Argon or nitrogen are saturated into the tube for 40 minutes and a 300 W Xenon lamp was used as the illumination source for the light samples. The samples were sealed under He and the EPR spectra were recorded at 4.2 K. All spectra are simulated using EasySpin software package Release 6.0.0-dev.26 (2020-10-12). (Stefan Stoll, Arthur Schweiger, EasySpin, a comprehensive software package for spectral simulation and analysis in EPR.[1]

In-Situ Infared Spectrascopy

In situ ATR measurements were performed using a Thermo Fisher iS20 equipped with an MCT/A detector. The Pike Technologies HATR accessory was used to investigate the reaction on the catalyst surface. The top of the HATR cell was modified in house with the addition of a quartz window to be able to perform photocatalytic experiments. In a typical experiment, a slurry was prepared by adding 2 ml of water to 15 mg of P25 and vigorously mixed until well dispersed. The slurry was then deposited drop-wise on a Ge refractive element (IRE) and the water was evaporated with a heat-gun. The coated IRE was inserted into the modified HATR module. A second IR spectrometer (Thermo Fisher iS5) equipped with a HATR cell using a ZnSe crystal was used to analyze the effluent of the iS20 to account for the contribution of the liquid phase. The solution of 5 vol% of MeOH was saturated with the gas of interest (Ar or N_2) for 40 min before being fed into the system. The solution was continuously purged with the gas for the length of the experiment to maintain saturation. The methanol solution was fed into the system at 0.5 ml min^{-1} for 30 minutes to stabilize

the flow. Afterwards, the Xenon lamp was turned on at 300 W. The acquisition of IR spectra was started on both the iS20 and the iS5 and the evolution of the spetra was followed for 350 minutes collecting a spectrum every 5 minutes by using a resolution of 4 cm^{-1} and 64 scans. The last spectrum collected in the absence of irradiation was used as background, and subtracted from the spectra obtained in the presence of irradiation to highlight the vibrations of the molecules bound to the catalyst surface.

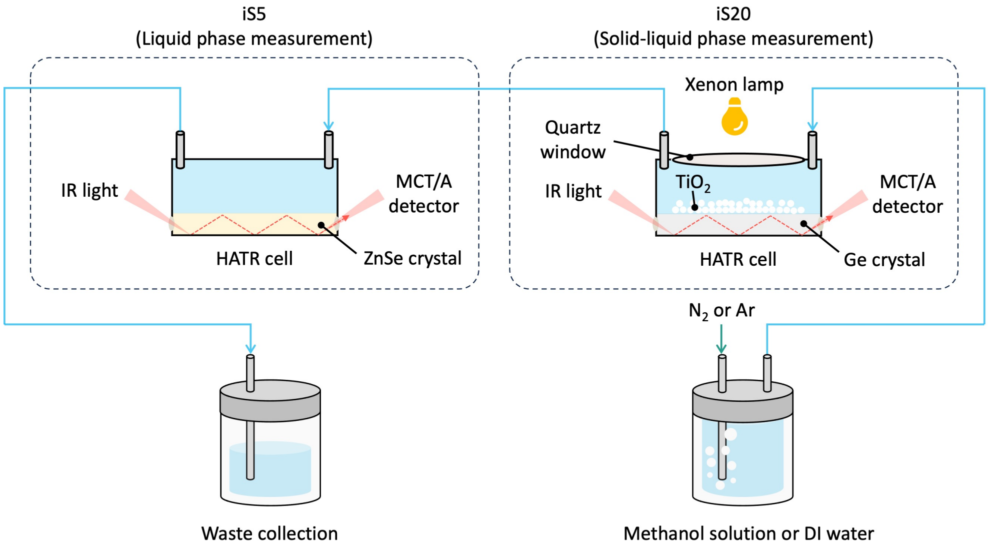

Figure S1: Schematic diagram of the in situ IR experiment.

Density Functional Theory

All stationary geometry optimization and generalized gradient approximation (GGA) functional calculations were performed in the Quantum ESPRESSO software package[2] together with the Atomic Simulation Package (ASE).[3] The Bayesian error estimation functional with van der Waals correlation (BEEF-vdW)[4] was used to describe electronic interactions. The plane-wave cutoff energy was set at 400 eV for all calculations; and a Monkhorst-Pack k-point grid spacing of $4 \times 4 \times 1$ was used for calculations. The NNIN/C pseudopotential[5] was chosen to treat the core-electron interactions. A dipole correction was applied for all slab calculations. All intermediate states were spin-paired except CH*. Adsorbate configurations

were determined by taking the most stable binding site from several high-symmetry configurations. When possible, this was achieved by using the most stable site from prior work, while for new adsorbates, configurations were manually enumerated and optimized with the BEEF-vdW functional to determine the most stable site. All geometries were optimized using the BFGS line search algorithm. The convergence threshold was set at 10^{-6}eV. Gas phase calculations were performed at the Γ point in a unit cell with 6 Å vacuum. The model surface was a 4 layers rutile (110) slab with one oxygen vacancy as the adsorption site.[6] Geometries of adsorbed surfaces were determined by trying adsorbates of multiple orientations and taking the lowest energy.

HSE06[7] hybrid functional calculations were performed in the Simulation Package for Ab-initio Real-space Calculations (SPARC) software package.[8] The Monkhorst-Pack k-point grid spacing of $4 \times 4 \times 1$ and a mesh spacing of 0.1 Å were used for calculations. For slab calculations, periodic and Dirichlet boundary conditions were prescribed in the plane and perpendicular to the plane of the slab, respectively. For gas phase molecules calculations, Dirichlet boundary conditions are employed in all three coordinate directions. All adsorbed intermediate states were calculated with spin polarization. The convergence tolerance on the normalized residual of electron density of the SCF iteration was set at 10^{-6}. The convergence tolerance on the Fock energy was set at 10^{-4} Hartree. The maximum number of Fock iterations was 20. The hybrid range screening parameter was set at 0.106.[7]

We utilized geometries reported by Xie et al[9] as the initial guesses of adsorbate geometries in the OH-assisted pathway on a periodic rutile TiO_2 (110) slab with oxygen vacancy (Per-slab) in Fig. S11 and S13. These states were relaxed with BEEF-vdW prior to a single-point calculation with HSE06. The relaxed geometries are shown in Table S5. The term Per-slab is used to distinguish from the oxygen vacancy site on a rutile TiO_2 (110) slab reported by Comer et al.[10]

The electronic energies for the reaction energies to form NH_3, N_2H_2, N_2H_4 and H_2O from N_2, H_2, and O_2 are compared for the BEEF-vdW and HSE functionals (Fig. S2).

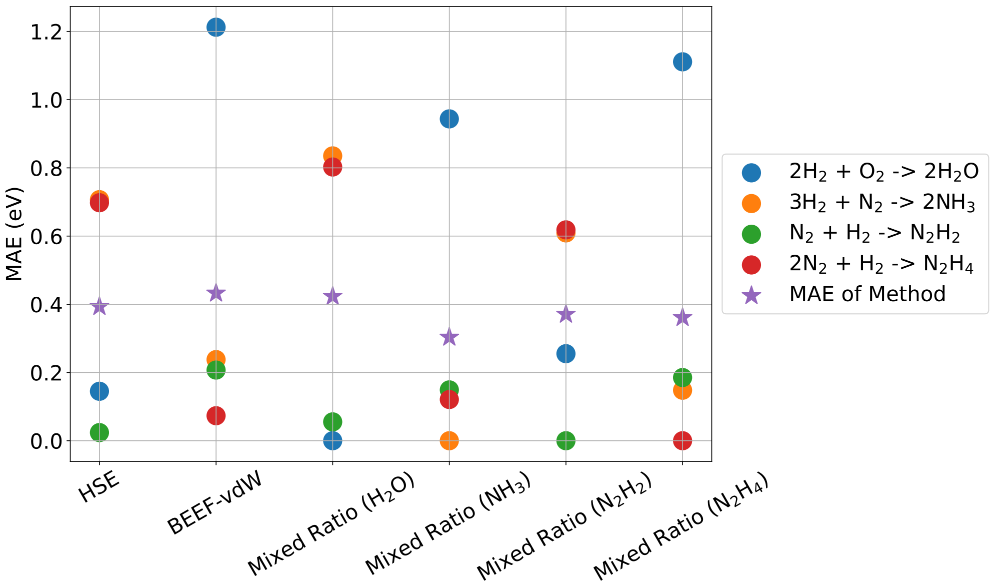

Figure S2: The scatter plot showing the mean absolute errors of reaction energy calculated from each method. The MAE values of each method are represented by stars. The x-axis represents the methods used, and the y-axis represents the MAE in electron volts (eV). The "Mixed Ratio (compound)" methods use a linear combination of BEEF-vdW and HSE energies to yield the experimental reaction energy of the compound.

Surprisingly, the error for HSE is the largest, with an error of 0.707 eV for the NH_3. The BEEF-vdW functional has a lower magnitude error of 0.238 eV, which is also in the opposite direction, suggesting that BEEF-vdW tends to systematically under-estimate N-H bond strength, while HSE tends to systematically over-estimate it. Given the importance of N-H bonding on all intermediates in the mechanism, we ensure that the NH_3 formation energy is correct by using a linear combination of BEEF-vdW and HSE energies for all states in the system. Conceptually this is equivalent to adjusting the ratio of Fock exchange, which is a common practice in hybrid functional design. A ratio of 0.748 BEEF-vdW and 0.252 HSE yields the correct NH_3 reaction energy, and reduces the mean absolute error of other relevant reaction energies to 0.303 eV from 0.433 eV (BEEF-vdW) or 0.393 eV (HSE). Thus, we expect that this combined energy provides the best estimate of intermediate states, and have used these energies in the main text. However, the qualitative conclusions reached do not depend on which functional is used, and the raw energies and free energy diagrams for both BEEF-vdW and HSE are provided below.

To include zero point energy (ZPE) and thermal contributions, vibrational frequency calculations and statistical mechanics corrections were performed using the ASE implementation. Then ground state electronic energies (E_{ele}) calculated by DFT were converted to free energies (G_i^o) using the following equation:

$$G_i^o = E_{ele} + E_{ZPE} + \Delta H - T\Delta S \tag{S1}$$

where E_{ZPE} is the zero point energy, ΔH and $T\Delta S$ are thermal contributions. Gas phase molecules were treated as ideal gas; and adsorbates were treated with the harmonic approximation with a low-frequency cutoff of 30 cm^{-1}. Relative free energies were computed with respect to reference states using the formula:

$$G_i = G_i^o - \sum_j n_j \mu_j \tag{S2}$$

where G_i is the free energy of species i, G_i^o is the total energy computed from DFT and free energy corrections, n_i is the number of atoms j in species i, and μ_j is the reference chemical potential. The reference for nitrogen was N_2 ($\mu_N = \frac{1}{2}G^o_{N_2}$), the reference for hydrogen was H_2 ($\mu_N = \frac{1}{2}G^o_{H_2}$), the reference for oxygen was H_2O ($\mu_O = G^o_{H_2O} - 2\mu_H$), and the reference for carbon was CH_3OH ($\mu_C = G^o_{CH_3OH} - 4\mu_H - \mu_O$).

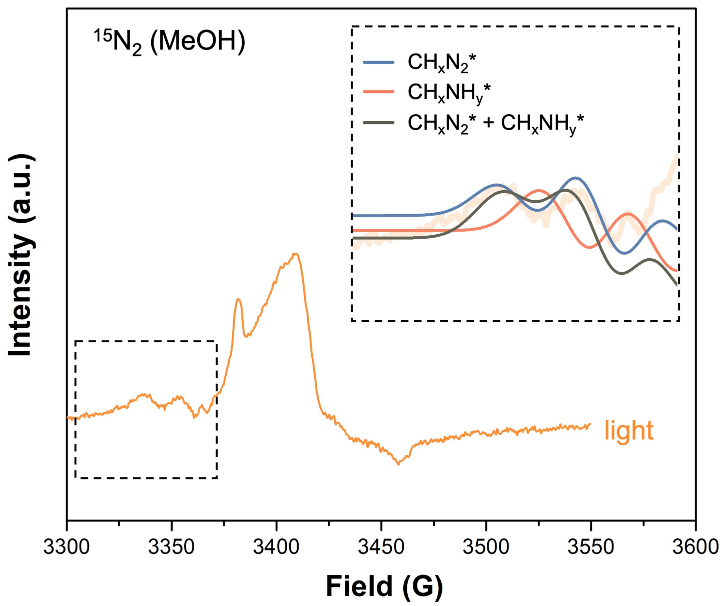

Figure S3: EPR spectra of titania with 5 vol% methanol solution at 4.2 K during illumination under $^{15}N_2$ nitrogen environment. Isotope ^{15}N has nuclear spin of 1/2 and therefore in $^{15}N_2$ environment the signal for nitrogen centered radical has 2 lines with the same intensity and $A_{15N} = 17G$ and he one for diazo centered radical is composed of 3 lines with line intensity 1:2:1 and hyperfine of $A_{15N} = 17G$.

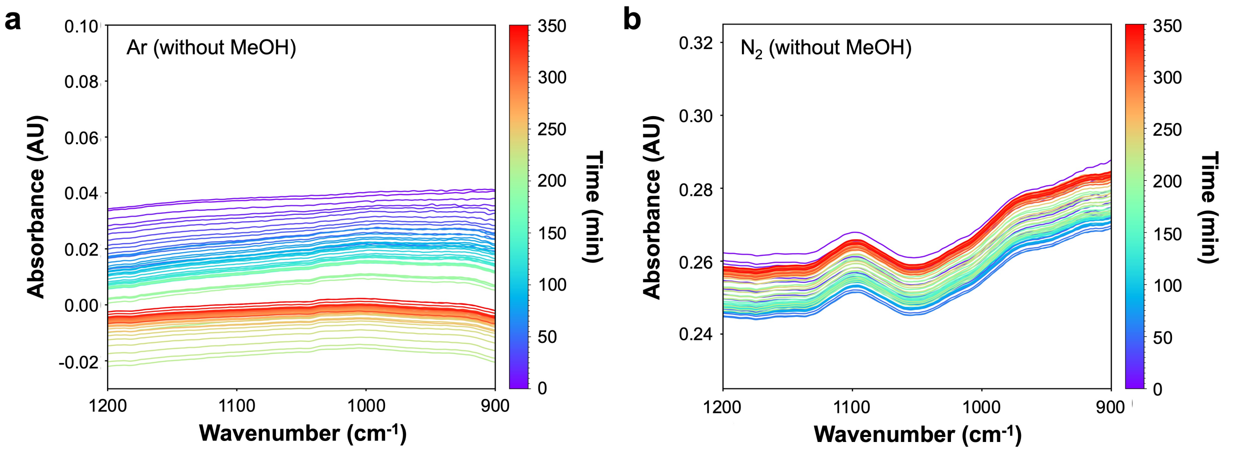

Figure S4: IR spectra of titania in water without methanol in (a) argon and (b) nitrogen environments during illumination.

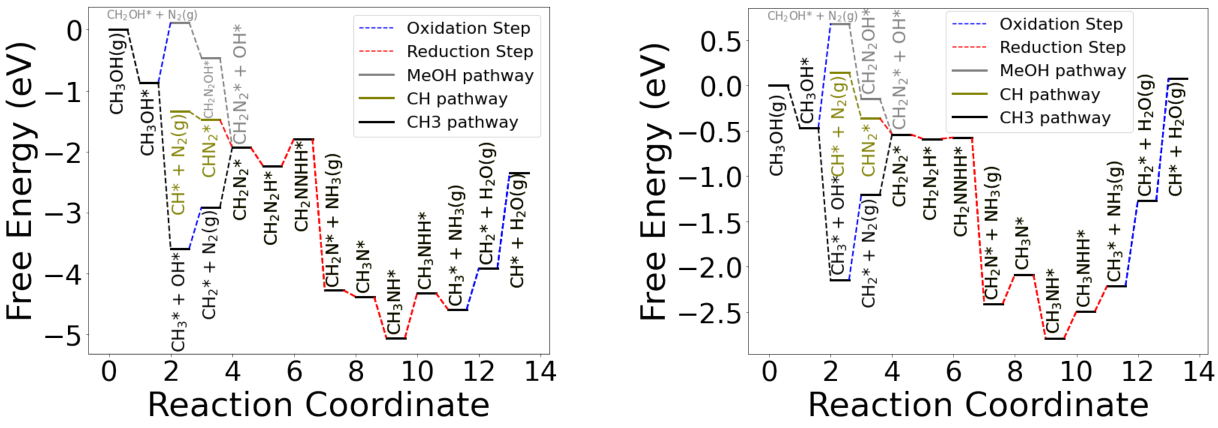

(a) (b)

Figure S5: Free energy diagram of all nitrogen fixation pathways at the oxygen-vacancy site. Zero is set to the energy of the oxygen-vacancy site. CH and CH_3 pathways energies were adjusted to ensure alignment with the methanol pathway calculated by HSE06 functional (a) and BEEF-vdW functional (b). The average standard deviation of the BEEF-vdW energy ensemble is $\pm$ 0.33 eV. Both diagrams include gas phase CH_3OH, H_2, N_2 and H_2O in the initial state.

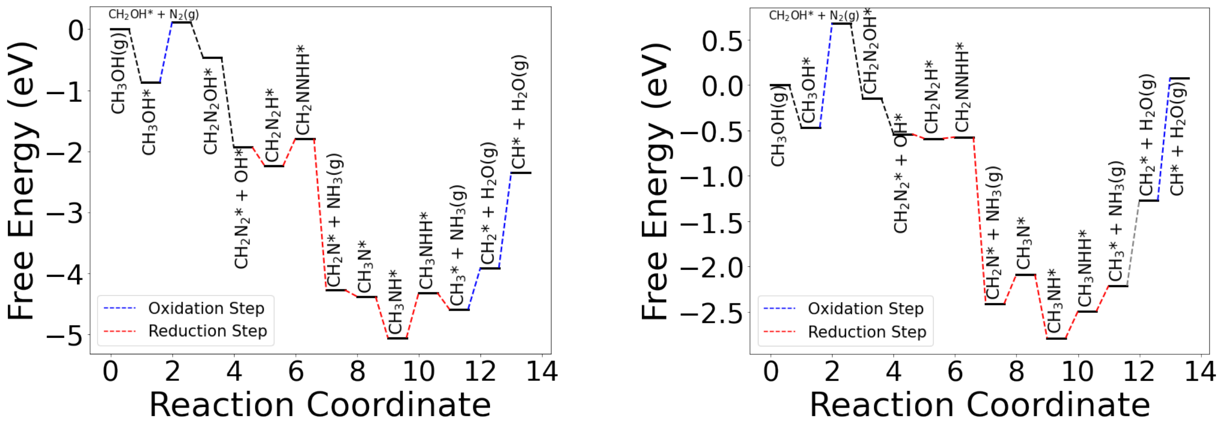

(a) (b)

Figure S6: Free energy diagram of nitrogen fixation starting from methanol at the oxygen-vacancy site calculated by HSE06 functional (a) and BEEF-vdW functional (b). Both diagrams include gas phase CH_3OH, H_2, N_2 and H_2O in the initial state.

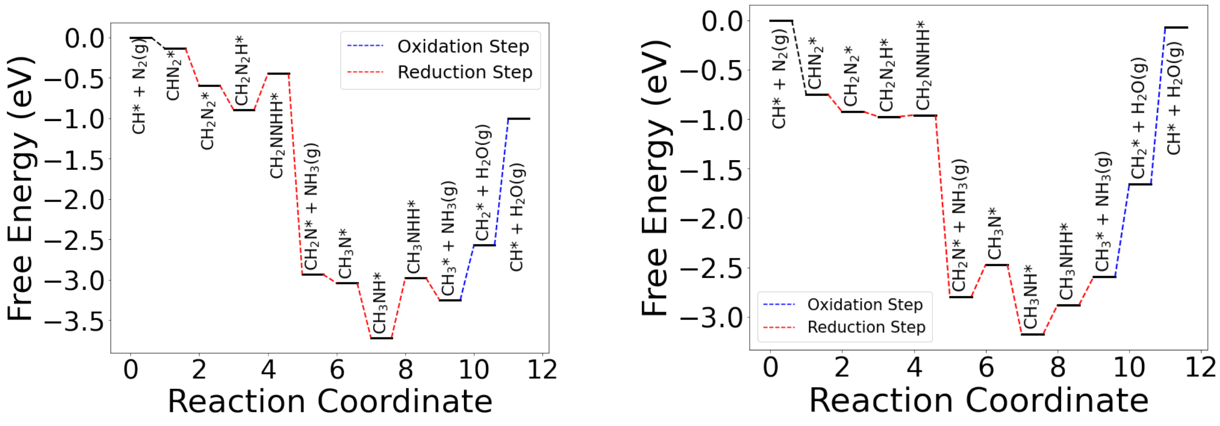

(a) (b)

Figure S7: Free energy diagram of nitrogen fixation starting from CH at the oxygen-vacancy site calculated by HSE06 functional (a) and BEEF-vdW functional (b). Both diagrams include gas phase H_2, N_2 and H_2O in the initial state.

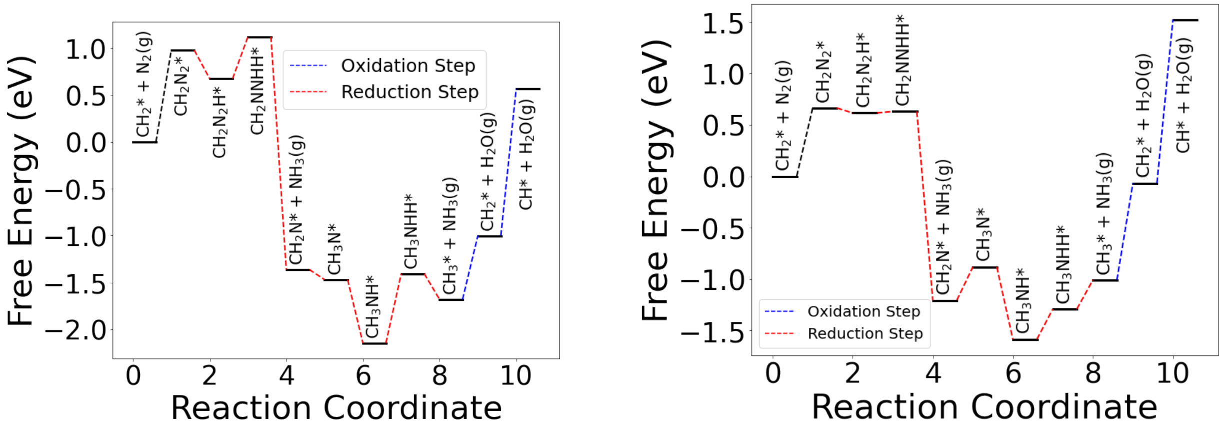

(a) (b)

Figure S8: Free energy diagram of nitrogen fixation starting from CH_2 at the oxygen-vacancy site calculated by HSE06 functional (a) and BEEF-vdW functional (b). Both diagrams include gas phase H_2, N_2 and H_2O in the initial state.

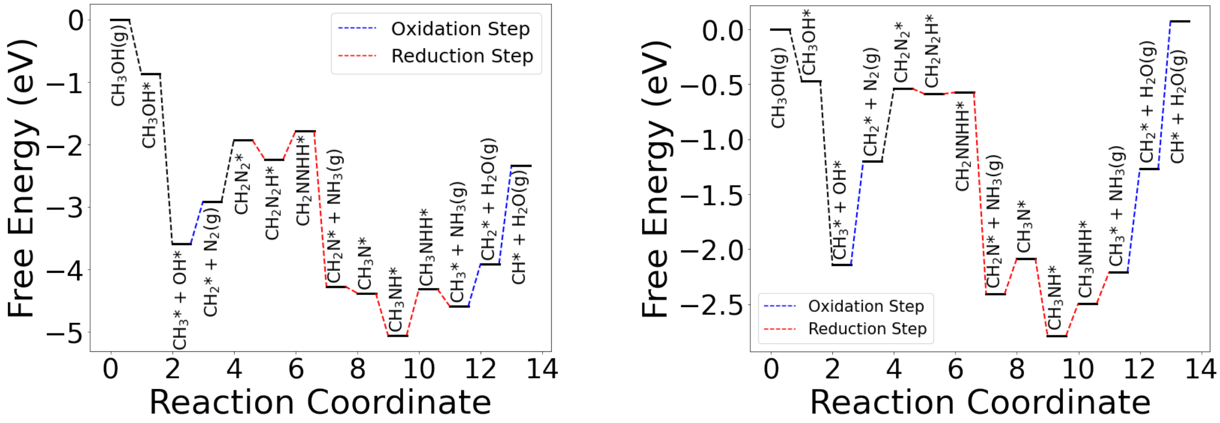

(a) (b)

Figure S9: Free energy diagram of nitrogen fixation starting from CH_3 at the oxygen-vacancy site calculated by HSE06 functional (a) and BEEF-vdW functional (b). Both diagrams include gas phase CH_3OH, H_2, N_2 and H_2O in the initial state.

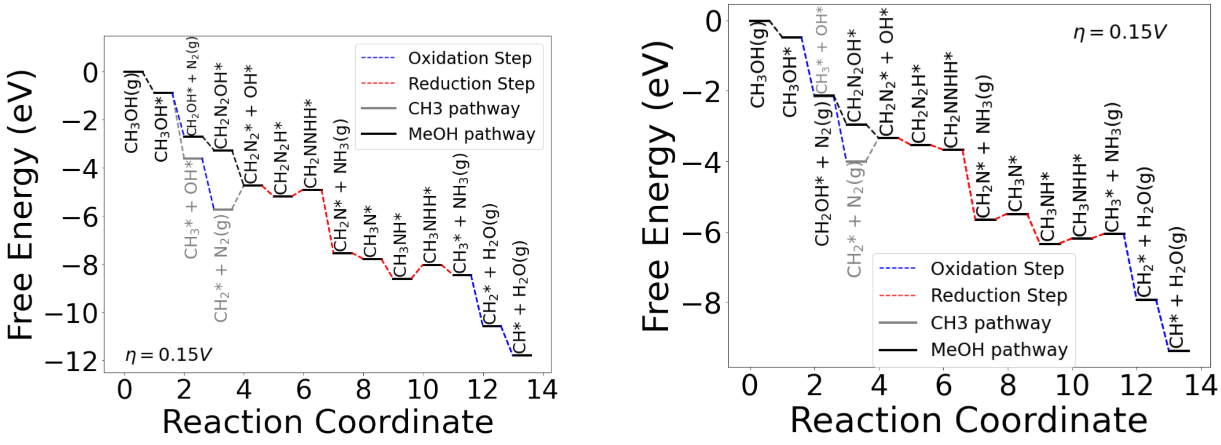

(a) (b)

Figure S10: Free energy diagram of nitrogen fixation starting from CH_3 and methanol at the oxygen-vacancy site at an overpotential (η) of 0.15V. calculated by HSE06 functional (a) and BEEF-vdW functional (b). Both diagrams include gas phase CH_3OH, H_2, N_2 and H_2O in the initial state.

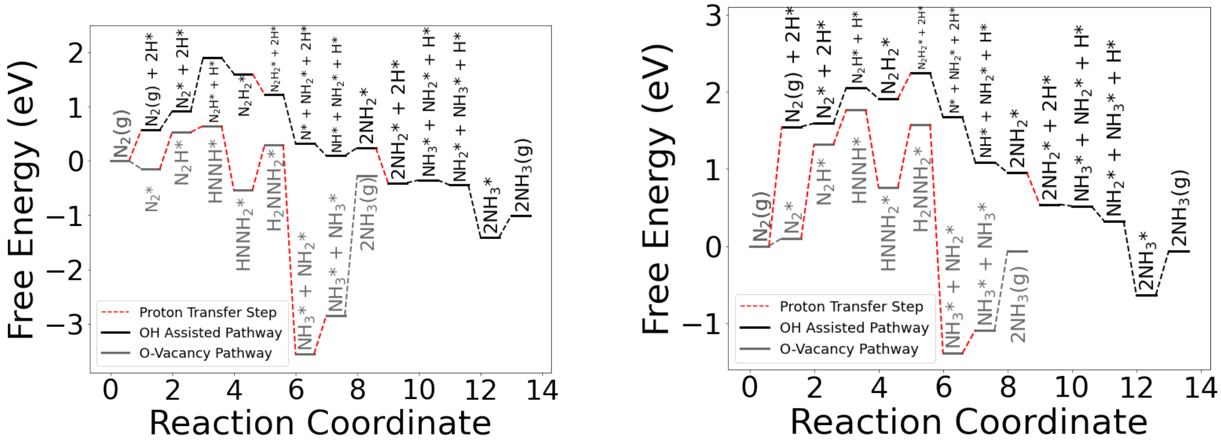

(a) (b)

Figure S11: Free energy diagram of nitrogen fixation pathways without carbon assistance starting from Per-slab (black) and the oxygen-vacancy site (grey), calculated by HSE06 functional (a) and BEEF-vdW functional (b). Both diagrams include gas phase H_2, N_2 and H_2O in the initial state.

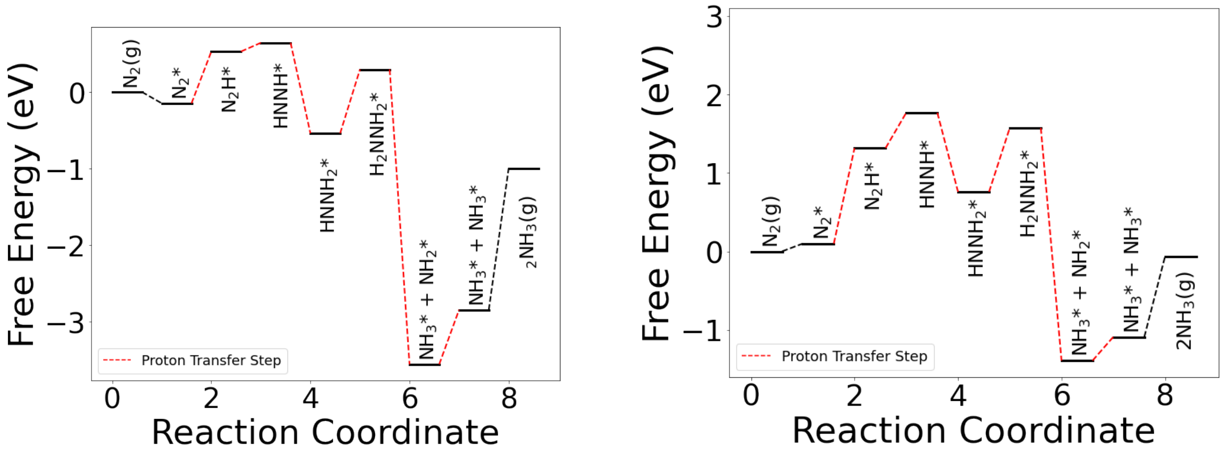

(a)

(b)

Figure S12: Free energy diagram of nitrogen fixation pathways without carbon assistance starting at the oxygen-vacancy site, calculated by HSE06 functional (a) and BEEF-vdW functional (b). Both diagrams include gas phase H_2, N_2 and H_2O in the initial state.

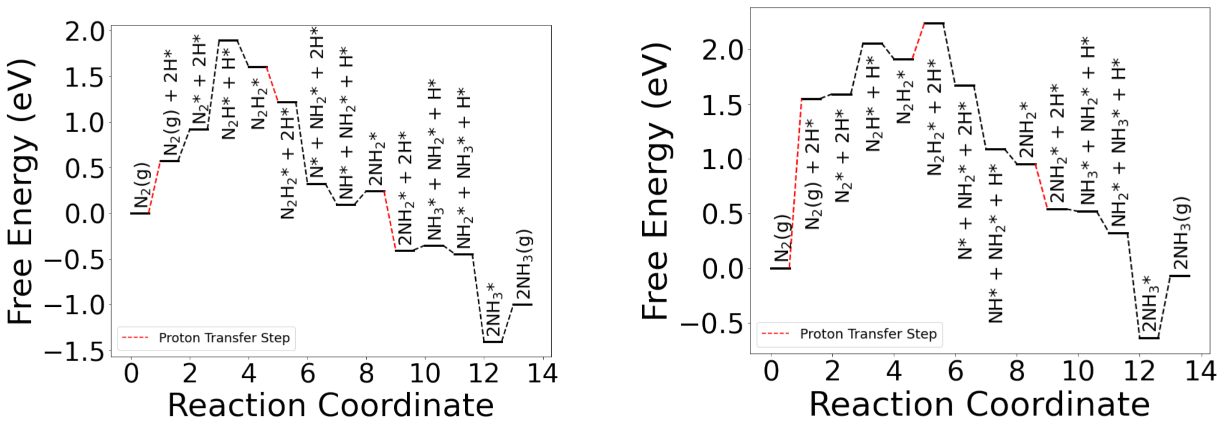

(a)

(b)

Figure S13: Free energy diagram of nitrogen fixation pathways without carbon assistance starting at the Per-slab, calculated by HSE06 functional (a) and BEEF-vdW functional (b). Both diagrams include gas phase H_2, N_2 and H_2O in the initial state.

Table S1: Energies of all species used relative to the slab with Oxygen vacancy at 0K (ZPE included)

compound	HSE06 energy	BEEF energy	ensemble standard deviation
CH_2N_2OH at Oxygen Vacancy	-0.05	0.26	0.38
CH_2N at Oxygen Vacancy	-0.86	-0.1	0.31
CHN_2 at Oxygen Vacancy	1.28	1.75	0.28
OH at Oxygen Vacancy	-2.33	-1.69	0.35
CH_3 at Oxygen Vacancy	-0.52	0.29	0.26
CH at Oxygen Vacancy	1.77	2.62	0.28
CH_3NH at Oxygen Vacancy	-1.68	-0.52	0.34
CH_2NNHH at Oxygen Vacancy	0.91	1.49	0.37
CH_2N_2H at Oxygen Vacancy	0.48	1.49	0.36
CH_2N_2 at Oxygen Vacancy	0.8	1.56	0.32
CH_3N at Oxygen Vacancy	-0.98	0.2	0.3
CH_3OH at Oxygen Vacancy	-0.12	0.28	0.37
CH_3NHH at Oxygen Vacancy	-0.96	-0.24	0.34
CH_2 at Oxygen Vacancy	0.18	1.25	0.24
CH_2OH at Oxygen Vacancy	0.89	1.45	0.4
N_2H at Oxygen Vacancy	0.15	0.94	0.33
NH_2 at Oxygen Vacancy	-2.35	-1.06	0.32
HNNH at Oxygen Vacancy	0.25	1.37	0.34

NH_3 at Oxygen Vacancy	-1.66	-0.78	0.31
$HNNH_2$ at Oxygen Vacancy	-0.95	0.34	0.39
N_2 at Oxygen Vacancy	-0.5	-0.26	0.18
H_2NNH_2 at Oxygen Vacancy	-0.14	1.14	0.45
CH_3OH gas	0.75	0.75	0.0
N_2 gas	-0.36	-0.36	0.0
NH_3 gas	-0.74	-0.27	0.13
H gas	-0.02	-0.02	0.0
H_2O gas	0.09	0.09	0.0

Table S3: Energies of all species used relative to the Per-slab with Oxygen vacancy at 0K (ZPE included)

compound	HSE06 energy	BEEF energy	ensemble standard deviation
$NH_2 + NH_3 + H$	-0.92	-0.15	0.57
$N_2H + H$	1.5	1.66	0.33
$2NH_2$	-0.19	0.52	0.4
$2NH_2 + 2H$	-0.88	0.07	0.58
N_2H_2	1.21	1.52	0.28
$NH_3 + NH_2 + H$	-0.82	0.05	0.59
2H	0.53	1.51	0.27
$N_2 + 2H$	0.52	1.19	0.35
$NH + NH_2 + H$	-0.34	0.65	0.4
$2NH_3$	-1.88	-1.11	0.59
$N + NH_2 + 2H$	-0.11	1.24	0.42
$N_2H_2 + 2H$	0.79	1.81	0.42

Table S4: Vibrational frequencies of all species

Compound	Vibrational Frequencies (cm^{-1})
CH_3OH gas	8.5, 12.9, 38.1<br>123.5, 132.1, 142.9<br>167.5, 181.0, 183.6<br>185.4, 365.7, 372.2<br>380.4, 473.4
N_2 gas	0.0, 0.0, 300.5
NH_3 gas	0.8, 2.0, 129.7<br>205.5, 205.5, 424.8<br>440.6, 440.7
H_2O gas	0.1, 3.0, 199.3<br>471.2, 485.3
CHN_2 at Oxygen Vacancy	4.9, 9.6, 22.5<br>36.4, 40.6, 63.4<br>70.3, 73.1, 143.0<br>146.2, 268.4, 381.4
CH at Oxygen Vacancy	16.3, 52.9, 60.4<br>69.0, 71.2, 380.8

CH_3NH at Oxygen Vacancy	15.3, 18.3, 34.8<br>45.5, 47.9, 78.6<br>127.3, 133.8, 139.3<br>165.5, 178.2, 182.6<br>184.9, 367.7, 374.6<br>376.9, 430.4
CH_2N_2 at Oxygen Vacancy	3.7, 5.6, 22.3<br>27.7, 35.7, 47.5<br>65.1, 91.0, 110.0<br>136.0, 142.9, 181.0<br>196.2, 373.8, 392.7
CH_2OH at Oxygen Vacancy	23.1, 24.4, 32.7<br>38.5, 53.0, 64.1<br>79.1, 89.7, 134.6<br>145.8, 152.9, 177.6<br>367.5, 377.4, 463.5
CH_3N at Oxygen Vacancy	7.3, 19.1, 39.5<br>47.1, 60.3, 132.2<br>133.9, 138.2, 175.5<br>180.5, 181.2, 362.1<br>367.7, 372.0

CH_2N at Oxygen Vacancy	10.8, 19.0, 39.0<br>40.7, 49.6, 56.8<br>133.3, 145.7, 182.1<br>208.9, 362.2, 370.3
CH_3NHH at Oxygen Vacancy	22.4, 28.9, 47.6<br>106.9, 123.9, 141.4<br>147.1, 170.6, 174.2<br>183.1, 187.7, 206.3<br>361.6, 372.1, 379.8<br>408.3, 425.9
CH_2N_2OH at Oxygen Vacancy	5.2, 12.4, 17.1<br>18.1, 21.7, 31.0<br>33.1, 35.5, 36.9<br>37.7, 39.1, 40.2<br>41.6, 42.4, 42.8<br>50.3, 50.9, 62.7<br>66.8, 85.0, 269.3
OH at Oxygen Vacancy	17.7, 35.7, 37.7<br>42.4, 47.8, 50.4

CH_3OH at Oxygen Vacancy	9.9, 10.8, 14.1<br>23.4, 28.5, 34.0<br>61.8, 121.8, 135.3<br>144.3, 165.9, 179.4<br>183.1, 183.2, 371.8<br>381.7, 387.1, 456.4
CH_2NNHH at Oxygen Vacancy	9.0, 10.8, 15.4<br>19.0, 26.5, 29.9<br>52.2, 68.7, 92.0<br>101.3, 114.7, 134.0<br>147.9, 166.8, 180.5<br>200.0, 207.2, 376.0<br>396.7, 415.6, 429.2
CH_2N_2H at Oxygen Vacancy	8.2, 20.1, 24.2<br>28.3, 39.8, 43.6<br>77.6, 82.9, 90.9<br>101.1, 136.4, 151.6<br>170.5, 178.0, 192.3<br>377.5, 397.5, 414.5
CH_2 at Oxygen Vacancy	26.3, 45.7, 47.5<br>64.0, 65.6, 75.0<br>160.7, 371.0, 381.6

CH_3 at Oxygen Vacancy	11.5, 23.0, 30.6<br>48.0, 73.4, 74.2<br>150.9, 175.0, 178.0<br>366.3, 375.5, 379.5
N_2H at Oxygen Vacancy	10.3, 20.3, 35.9<br>41.3, 44.8, 80.1<br>171.0, 198.0, 368.8
$HNNH_2$ at Oxygen Vacancy	14.6, 16.5, 24.5<br>32.3, 47.7, 48.7<br>81.4, 105.7, 133.4<br>157.1, 170.9, 203.7<br>419.8, 424.8, 432.5
N_2 at Oxygen Vacancy	4.3, 7.7, 9.4<br>14.9, 18.5, 299.3
HNNH at Oxygen Vacancy	12.1, 14.3, 24.5<br>31.5, 45.0, 46.9<br>115.5, 153.1, 165.7<br>185.2, 403.5, 409.8

H_2NNH_2 at Oxygen Vacancy	16.0, 20.2, 31.2<br>32.3, 41.5, 61.9<br>65.5, 116.0, 121.1<br>135.1, 141.6, 166.9<br>199.7, 202.7, 419.9<br>420.7, 434.0, 436.9
NH_2 at Oxygen Vacancy	29.7, 45.1, 54.1<br>69.9, 75.4, 84.7<br>191.1, 428.0, 438.7
$NH_2 + NH_3 + H$ at Per-slab with Oxygen Vacancy	14.5, 18.5, 22.2<br>25.4, 32.9, 41.5<br>61.9, 64.6, 67.7<br>68.6, 70.2, 139.9<br>190.2, 201.4, 202.8<br>423.4, 430.6, 439.8<br>440.4, 442.7, 472.5
$N_2H + H$ at Per-slab with Oxygen Vacancy	10.1, 16.6, 37.9<br>42.0, 45.7, 50.4<br>68.2, 84.2, 170.7<br>194.7, 371.3, 450.9

$2NH_2$ at Per-slab with Oxygen Vacancy	15.1, 22.1, 25.3<br>28.2, 37.6, 55.6<br>60.5, 67.5, 70.4<br>74.9, 187.3, 194.3<br>423.0, 431.7, 433.6<br>445.0
$2NH_2$ + 2H at Per-slab with Oxygen Vacancy	17.9, 21.8, 29.1<br>37.1, 42.8, 53.8<br>57.0, 59.7, 63.4<br>68.8, 73.7, 76.3<br>76.8, 82.1, 120.6<br>134.5, 189.1, 190.3<br>321.7, 425.5, 428.2<br>438.1, 440.0, 461.9
N_2H_2 at Per-slab with Oxygen Vacancy	10.1, 16.6, 39.8<br>43.2, 52.0, 55.7<br>63.7, 154.1, 164.7<br>200.0, 415.6, 432.0

NH_3 + NH_2 + H at Per-slab with Oxygen Vacancy	15.5, 17.4, 19.4<br>21.7, 26.4, 34.8<br>63.5, 65.1, 66.4<br>67.7, 75.8, 143.3<br>187.7, 200.7, 203.1<br>423.6, 430.1, 439.3<br>439.7, 443.2, 469.7
2H at Per-slab with Oxygen Vacancy	70.8, 79.4, 462.6<br>463.9
N_2 + 2H at Per-slab with Oxygen Vacancy	3.0, 5.9, 7.2<br>9.7, 10.7, 60.7<br>63.6, 82.1, 89.0<br>297.2, 457.6, 459.6
NH + NH_2 + H at Per-slab with Oxygen Vacancy	14.3, 21.5, 26.4<br>34.4, 49.0, 50.4<br>66.4, 67.1, 70.0<br>75.9, 85.1, 186.9<br>432.1, 435.1, 445.6<br>469.5

$2NH_3$ at Per-slab with Oxygen Vacancy	14.4, 14.8, 15.1<br>17.7, 17.7, 24.1<br>30.6, 60.2, 61.7<br>66.2, 67.6, 139.0<br>142.1, 201.5, 202.8<br>202.9, 203.8, 418.9<br>423.7, 434.9, 439.7<br>439.8, 441.2
N + NH_2 + 2H at Per-slab with Oxygen Vacancy	10.8, 19.1, 21.6<br>37.8, 41.6, 67.2<br>67.7, 69.0, 80.8<br>86.8, 88.4, 186.6<br>431.6, 445.1, 468.4<br>470.8
N_2H_2 + 2H at Per-slab with Oxygen Vacancy	7.0, 21.5, 34.5<br>40.8, 47.7, 51.7<br>61.4, 67.0, 77.0<br>84.4, 94.3, 151.6<br>156.7, 199.7, 415.7<br>428.3, 461.8, 463.3

Table S5: Calculated equilibrium geometries for all species.

Compound	Top View	Side View
CHN_2 at Oxygen Vacancy	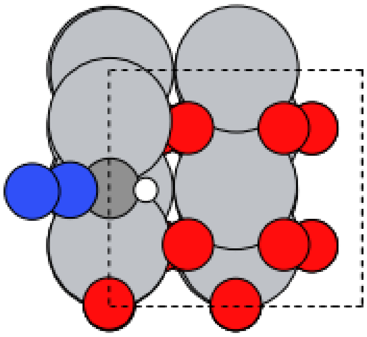	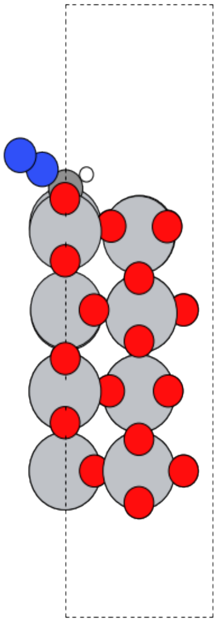
CH at Oxygen Vacancy	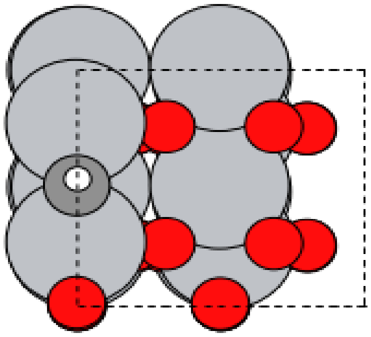	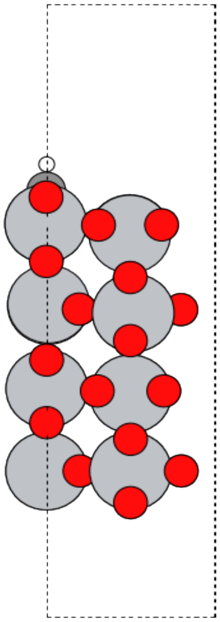

CH_3NH at Oxygen Vacancy	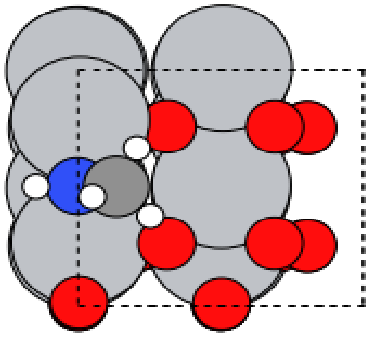	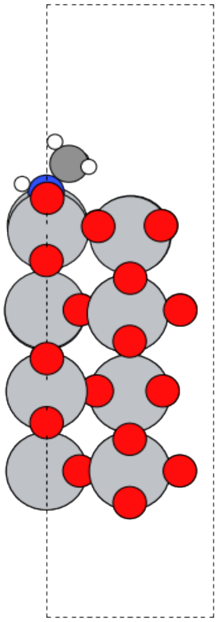
CH_2N_2 at Oxygen Vacancy	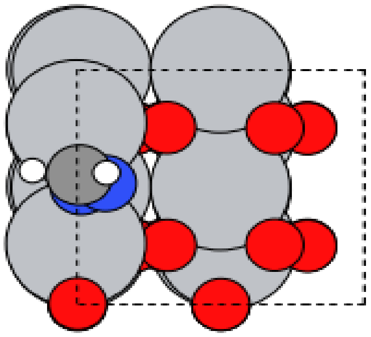	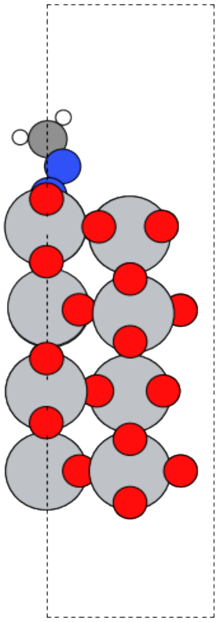

CH_2OH at Oxygen Vacancy	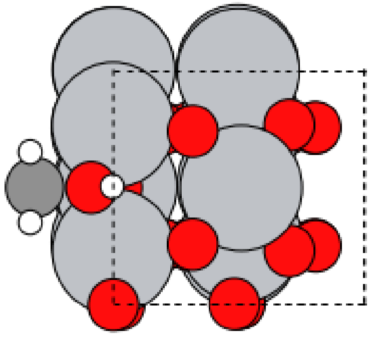	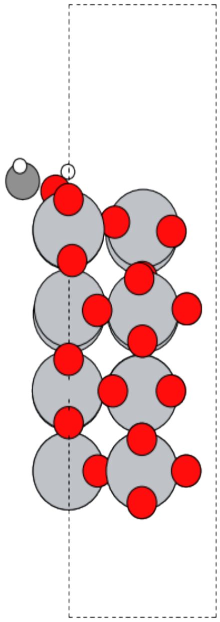
CH_3N at Oxygen Vacancy	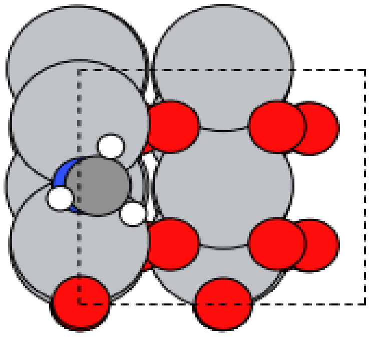	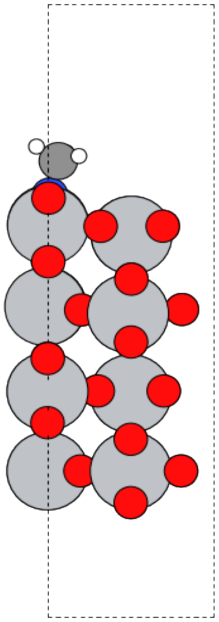

CH_2N at Oxygen Vacancy	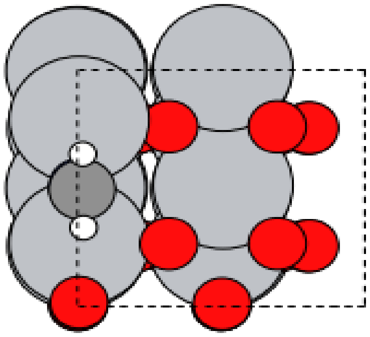	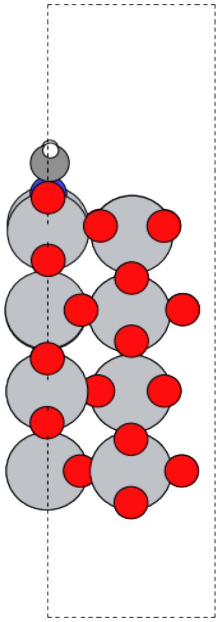
CH_3NHH at Oxygen Vacancy	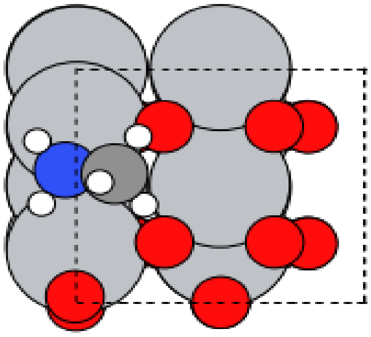	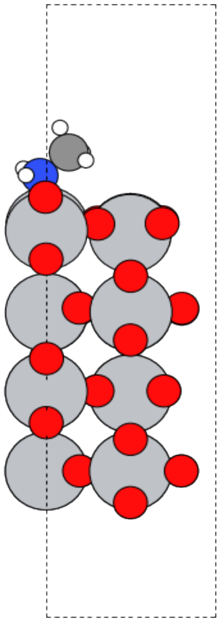

CH_2N_2OH at Oxygen Vacancy	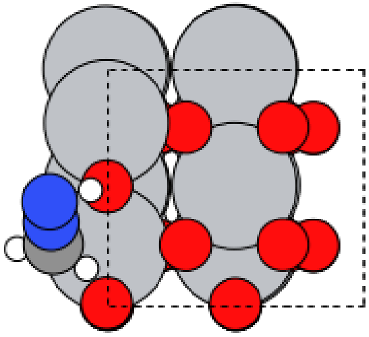	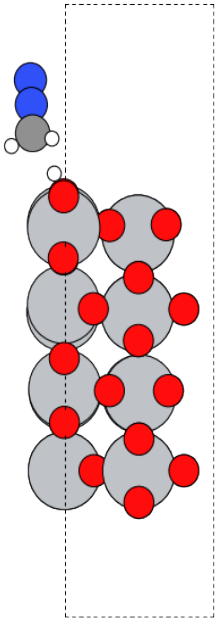
OH at Oxygen Vacancy	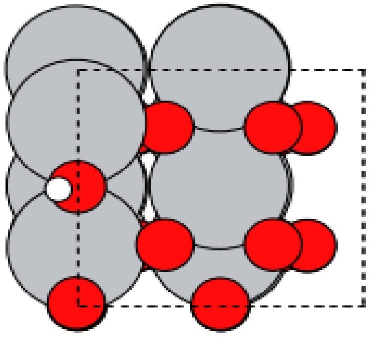	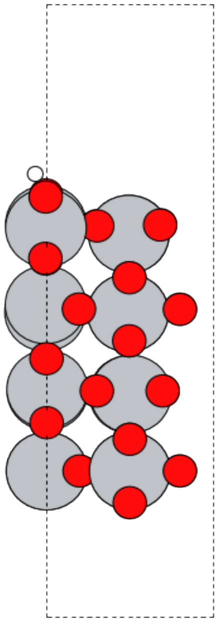

CH_3OH at Oxygen Vacancy	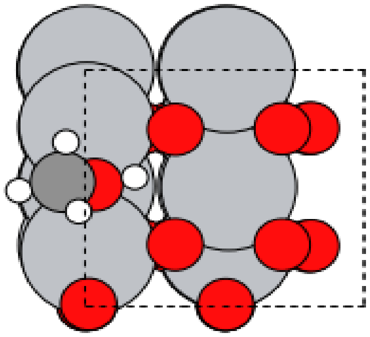	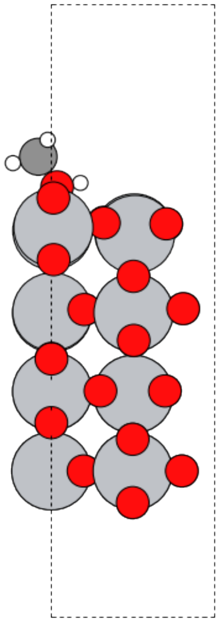
CH_2NNHH at Oxygen Vacancy	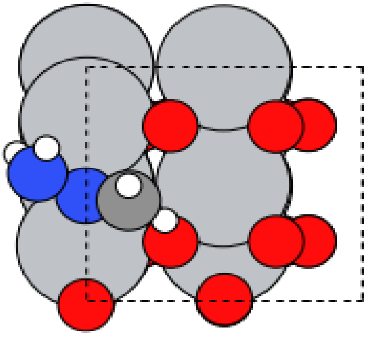	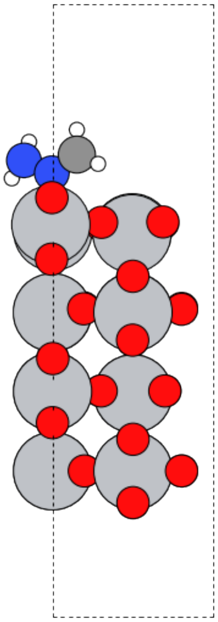

CH_2N_2H at Oxygen Vacancy	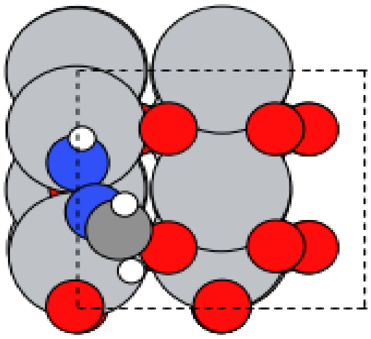	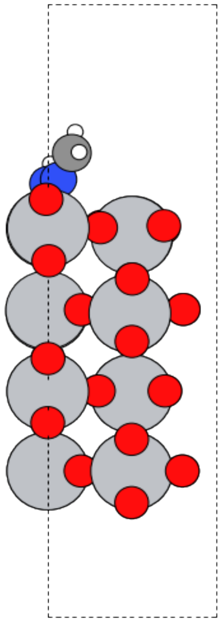
CH_2 at Oxygen Vacancy	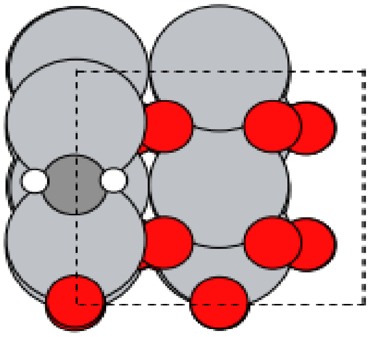	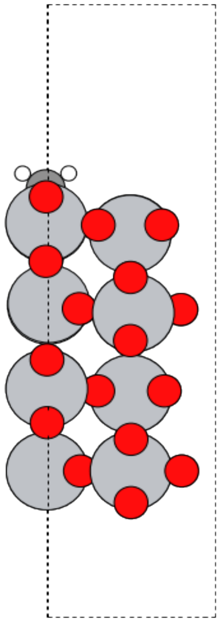

CH_3 at Oxygen Vacancy	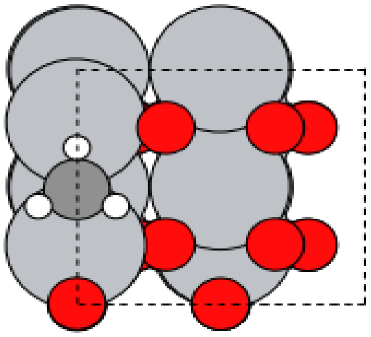	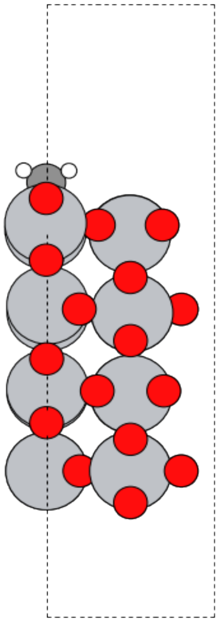
N_2H at Oxygen Vacancy	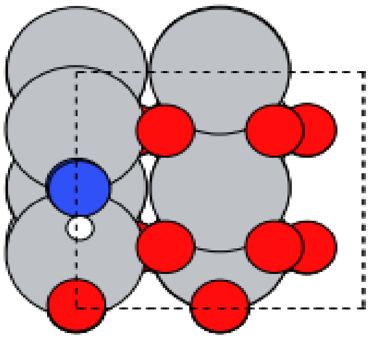	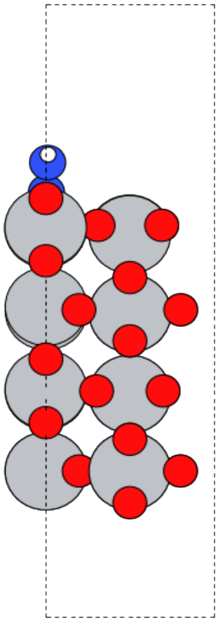

$HNNH_2$ at Oxygen Vacancy	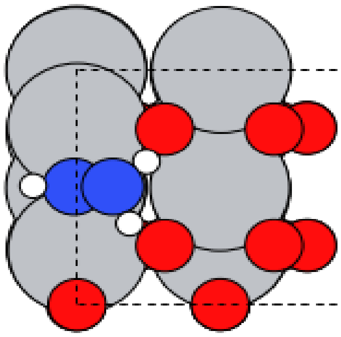	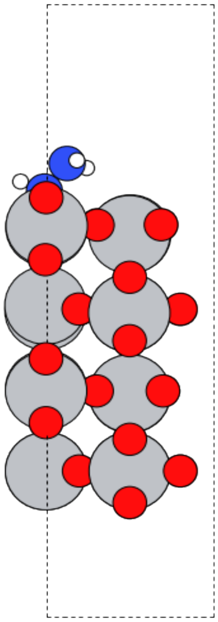
N_2 at Oxygen Vacancy	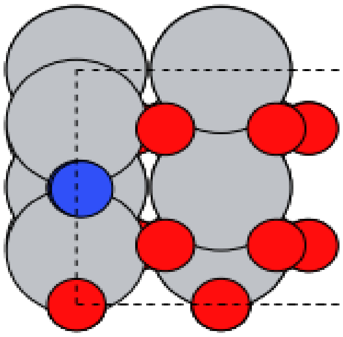	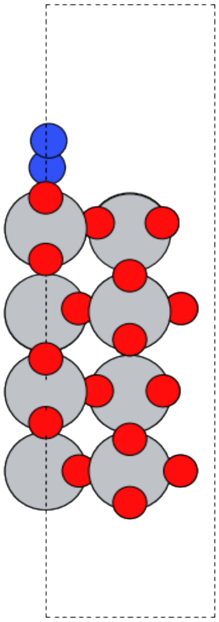

HNNH at Oxygen Vacancy	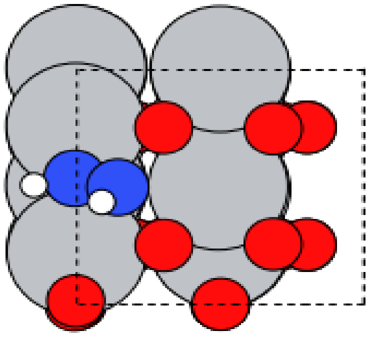	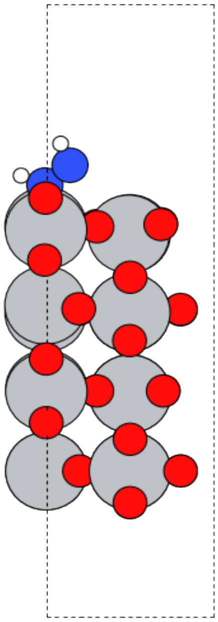
H_2NNH_2 at Oxygen Vacancy	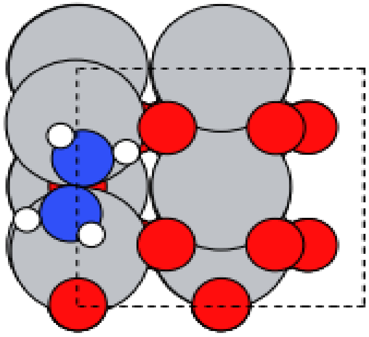	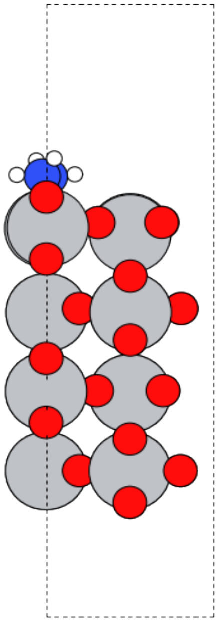

NH_3 at Oxygen Vacancy	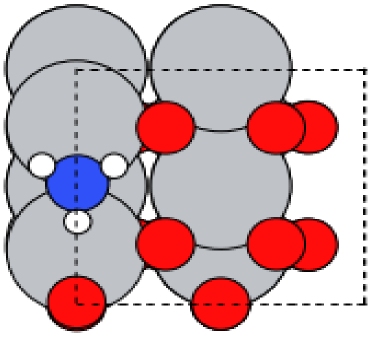	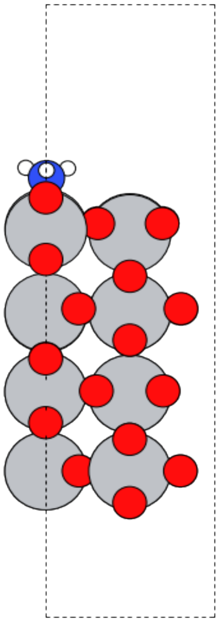
NH_2 at Oxygen Vacancy	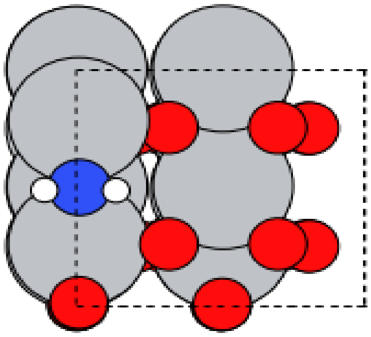	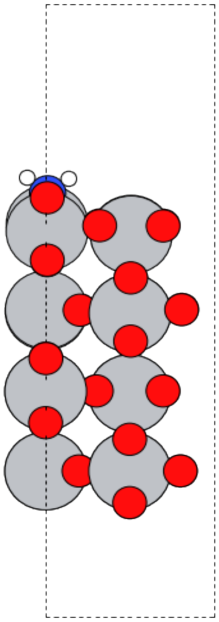

Rutile (110) with Oxygen Vacancy	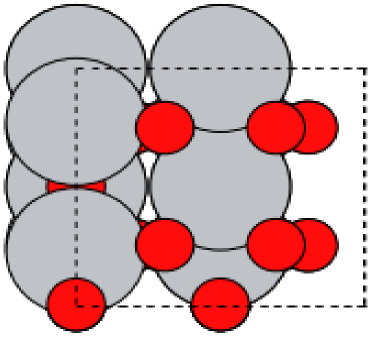	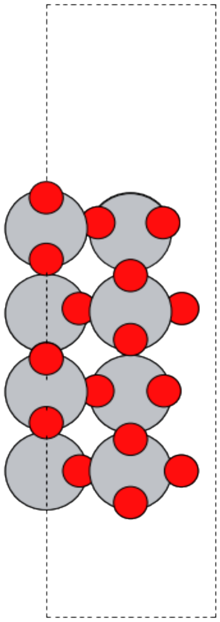
NH_2 + NH_3 + H at Per-slab with Oxygen Vacancy	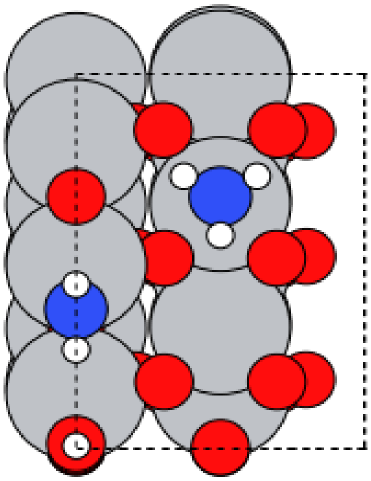	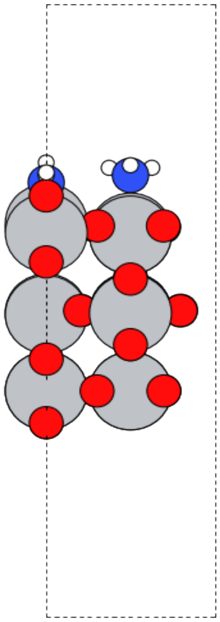

$N_2H + H$ at Per-slab with Oxygen Vacancy	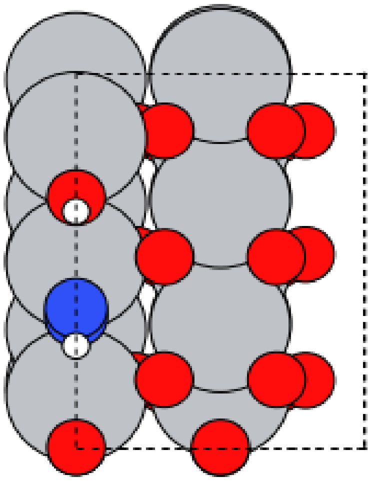	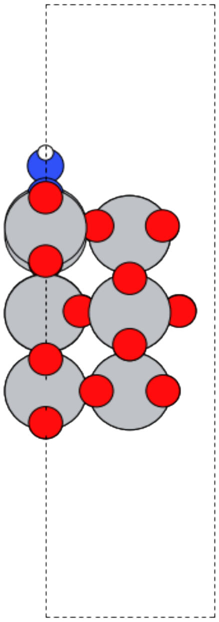
$2NH_2$ at Per-slab with Oxygen Vacancy	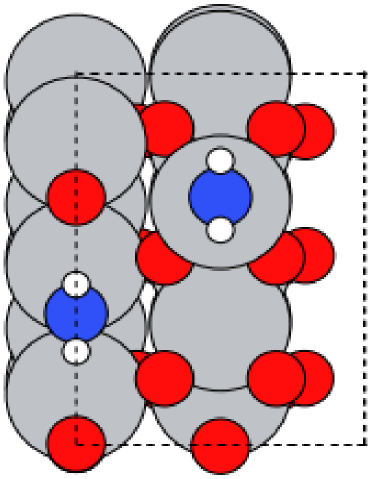	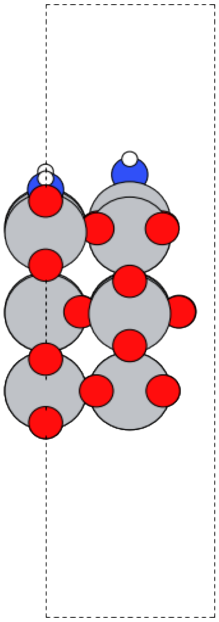

$2NH_2$ + 2H at Per-slab with Oxygen Vacancy	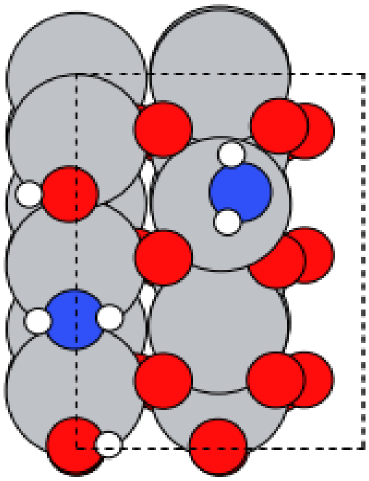	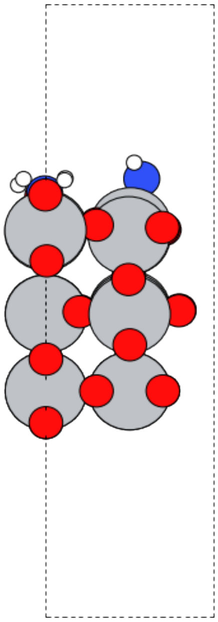
N_2H_2 at Per-slab with Oxygen Vacancy	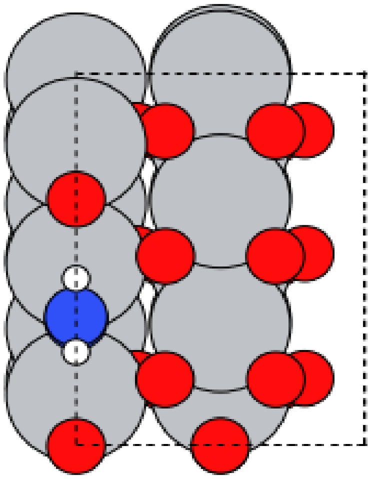	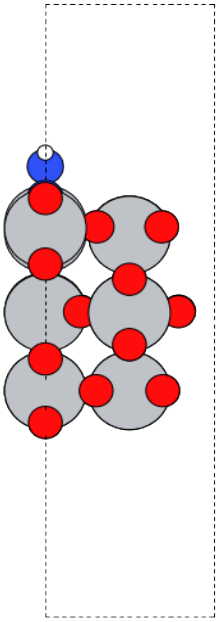

NH_3 + NH_2 + H at Per-slab with Oxygen Vacancy	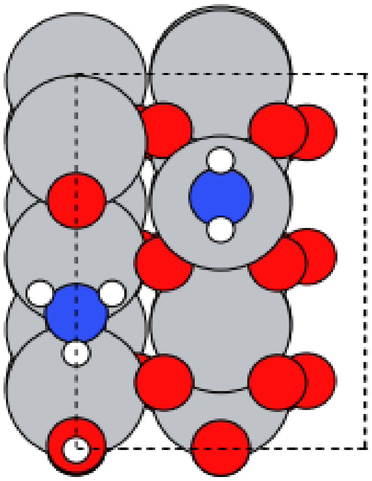	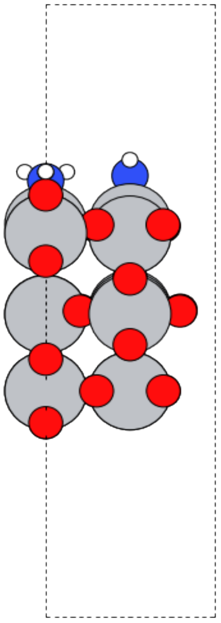
2H at Per-slab with Oxygen Vacancy	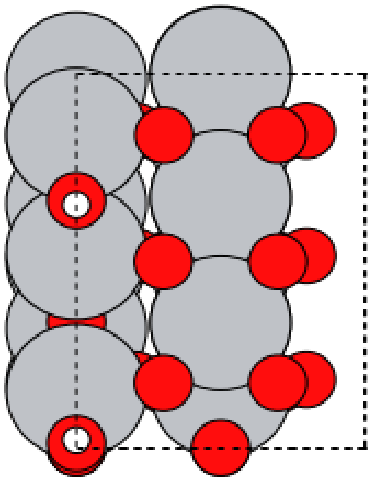	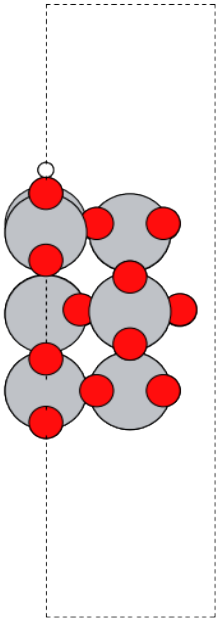

N_2 + 2H at Per-slab with Oxygen Vacancy	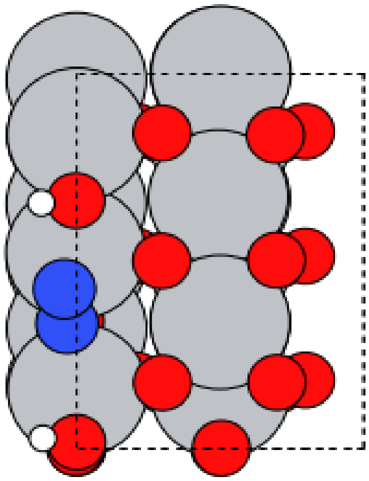	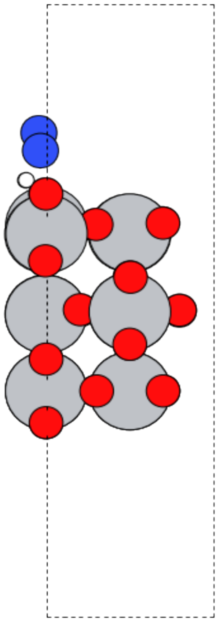
NH + NH_2 + H at Per-slab with Oxygen Vacancy	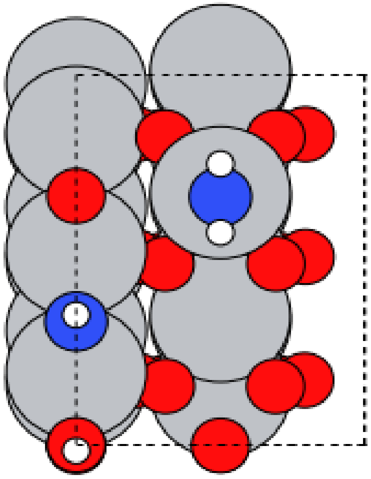	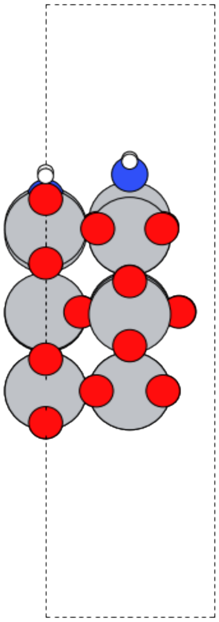

$2NH_3$ at Per-slab with Oxygen Vacancy	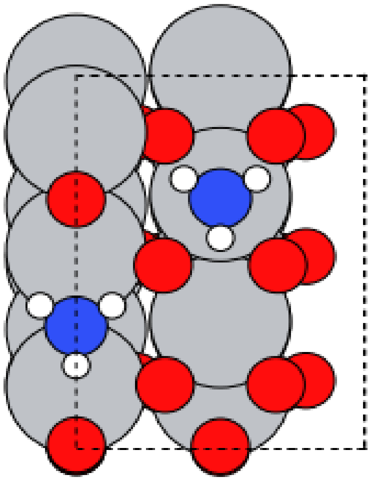	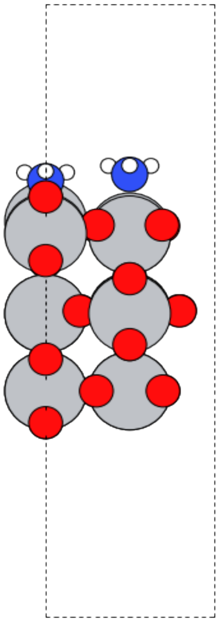
$N + NH_2 + 2H$ at Per-slab with Oxygen Vacancy	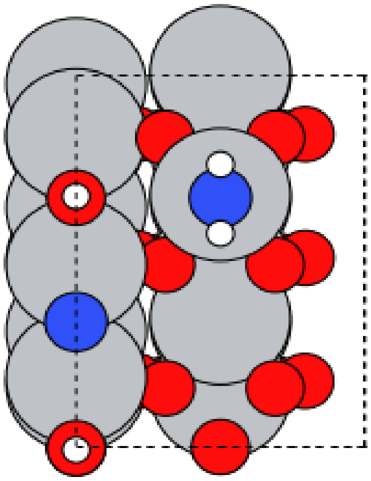	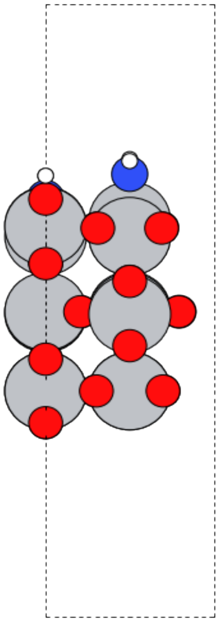

N_2H_2 + 2H at Per-slab with Oxygen Vacancy	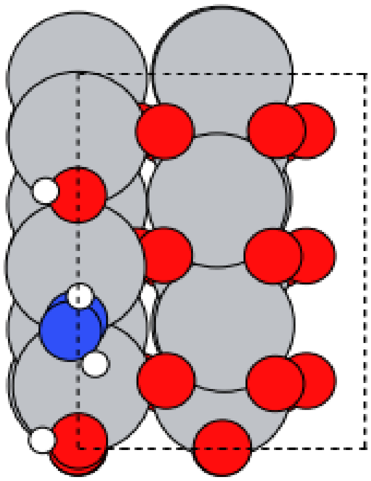	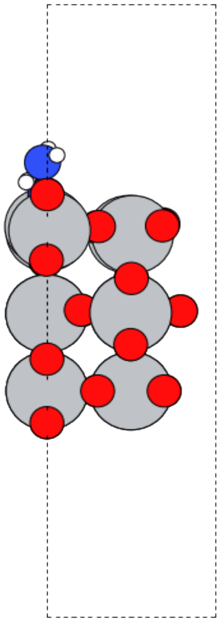
Per-slab with Oxygen Vacancy	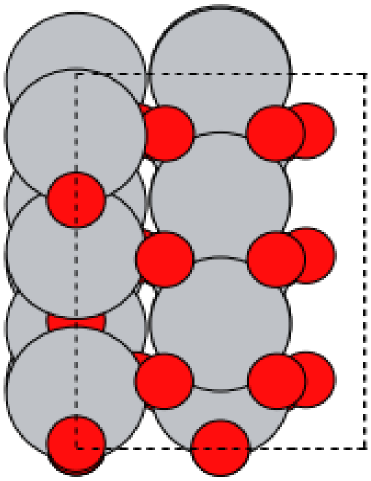	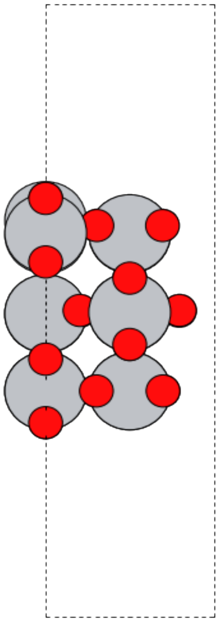

References

(1) Stoll, S.; Schweiger, A. EasySpin, a comprehensive software package for spectral simulation and analysis in EPR. *Journal of magnetic resonance* **2006**, *178*, 42–55.

(2) Giannozzi, P.; Baroni, S.; Bonini, N.; Calandra, M.; Car, R.; Cavazzoni, C.; Ceresoli, D.; Chiarotti, G. L.; Cococcioni, M.; Dabo, I.; others QUANTUM ESPRESSO: a modular and open-source software project for quantum simulations of materials. *Journal of physics: Condensed matter* **2009**, *21*, 395502.

(3) Larsen, A. H.; Mortensen, J. J.; Blomqvist, J.; Castelli, I. E.; Christensen, R.; Dułak, M.; Friis, J.; Groves, M. N.; Hammer, B.; Hargus, C.; others The atomic simulation environment—a Python library for working with atoms. *Journal of Physics: Condensed Matter* **2017**, *29*, 273002.

(4) Wellendorff, J.; Lundgaard, K. T.; Møgelhøj, A.; Petzold, V.; Landis, D. D.; Nørskov, J. K.; Bligaard, T.; Jacobsen, K. W. Density functionals for surface science: Exchange-correlation model development with Bayesian error estimation. *Physical Review B* **2012**, *85*, 235149.

(5) `http://nninc.cnf.cornell.edu/`.

(6) Comer, B. M.; Liu, Y.-H.; Dixit, M. B.; Hatzell, K. B.; Ye, Y.; Crumlin, E. J.; Hatzell, M. C.; Medford, A. J. The role of adventitious carbon in photo-catalytic nitrogen fixation by titania. *Journal of the American Chemical Society* **2018**, *140*, 15157–15160.

(7) Heyd, J.; Scuseria, G. E.; Ernzerhof, M. Hybrid functionals based on a screened Coulomb potential. *The Journal of Chemical Physics* **2003**, *118*, 8207–8215.

(8) Ghosh, S.; Suryanarayana, P. SPARC: Accurate and efficient finite-difference formu-

lation and parallel implementation of Density Functional Theory: Extended systems. *Computer Physics Communications* **2017**, *216*, 109–125.

(9) Xie, X.-Y.; Xiao, P.; Fang, W.-H.; Cui, G.; Thiel, W. Probing Photocatalytic Nitrogen Reduction to Ammonia with Water on the Rutile TiO2 (110) Surface by First-Principles Calculations. *ACS Catalysis* **2019**, *9*, 9178–9187.

(10) Comer, B. M.; Medford, A. J. Analysis of photocatalytic nitrogen fixation on rutile TiO2 (110). *ACS Sustainable Chemistry & Engineering* **2018**, *6*, 4648–4660.
